# Reinforcement learning-controlled differential evolution with L-BFGS refinements

**DOI:** 10.1371/journal.pone.0347860

**Published:** 2026-05-13

**Authors:** Yang Cao, Bingchuan Wu, Miao Wen

**Affiliations:** 1 School of Computer Science and Engineering, Shenyang Jianzhu University, Shenyang, China; 2 Liaoning Provincial Key Laboratory of Big Data Management and Analysis of Urban Construction, Shenyang, China; 3 Shenyang Branch of National Special Computer Engineering Technology Research Center, Shenyang, China; Hiroshima University: Hiroshima Daigaku, JAPAN

## Abstract

Differential evolution has become one of the mainstream solvers for complex optimization problems due to its concise structure and strong global search ability. However, the performance of the DE algorithm is highly sensitive to its mutation and crossover strategies and related control parameters. Traditional adaptive strategies are mostly based on heuristic rules and lack online learning capabilities, which limits their further application in expensive black box optimization scenarios. Therefore, this article proposes Reinforcement Learning-controlled Differential Evolution with Limited-memory Broyden-Fletcher-Goldfarb-Shanno Refinement (RL-DE). The algorithm has constructed a three-layer closed-loop framework of “parameters policy refinement”, achieving a paradigm shift from rule driven to data-driven. The system evaluation on the Congress on Evolutionary Computation 2017 (CEC2017) benchmark test set shows that the algorithm is robust in high-dimensional scenarios and achieves better results compared to classical adaptive DE variants; Its successful application in flexible job shop scheduling problems also validates the generalization ability of the framework towards the field of discrete combinatorial optimization. This study provides a learnable unified architecture for parameter adaptation in DE, offering efficient and intelligent solutions for expensive black box optimization and engineering scheduling problems.

## 1. Introduction

Differential Evolution (DE), as a population-based stochastic optimization algorithm, has established its efficiency and wide application in solving complex, nonlinear, and non convex black box optimization problems [1]. Its success is attributed to the simple yet powerful mechanism of using vector differences to perturb the population, which gives it a strong ability to explore the global search space [2]. However, the performance of the standard DE algorithm is greatly influenced by its control parameter settings [3], including population size (NP), mutation factor (F), and crossover probability (CR). The optimal configuration of these parameters not only depends on the specific problem, but may also vary during different optimization stages of solving the same problem. Finding a universally applicable optimal parameter setting is a very difficult and time-consuming task, often requiring a lot of trial and error work, which weakens the black box characteristics of the algorithm. This dependency has prompted extensive research to focus on developing adaptive or self-adjusting mechanisms to dynamically adjust these parameters during the search process. Although these methods have shown improvement, many of them rely on fixed, predefined rules or heuristic methods, which may not be flexible enough to adapt to the changing terrain in complex optimization problems. The challenge lies in creating a parameter control mechanism that can intelligently learn and adapt to the specific characteristics of the problem at hand in real-time, thereby maximizing the efficiency and robustness of the algorithm.

Reinforcement Learning (RL), as a powerful paradigm for solving sequential decision problems, provides a systematic framework for solving sequential decision problems, in which agents learn to make optimal decisions by interacting with the environment to maximize cumulative rewards [4]. This paradigm has been widely applied in the field of optimization, especially in the automation design and control of metaheuristic algorithms [5]. The core idea is to model the algorithm configuration or parameter control process as a Markov Decision Process (MDP), where the state represents the current state of the optimization process (such as population diversity, fitness terrain features), the action corresponds to the adjustment of algorithm parameters, and the reward comes from the improvement of solution quality or other performance indicators [6]. By using reinforcement learning techniques such as Q-learning [7] or policy gradient methods, agents can be trained to learn a parameter controlled optimal policy that dynamically adapts to the constantly evolving search terrain [8,9]. Compared to the discretization limitations of Q-learning, the policy gradient method can directly optimize the continuous parameter distribution, avoiding the curse of dimensionality, and is particularly suitable for continuous control problems such as DE parameters. This approach goes beyond static or heuristic based rules, allowing algorithms to learn optimization from experience, whether on specific problems or on a set of training problems. Integrating reinforcement learning into evolutionary algorithms such as differential evolution (DE) provides a promising direction for creating smarter and more autonomous optimization systems that can self adjust parameters to achieve superior performance without human intervention.

In recent years, it has been proven that reward shaping and curriculum learning can alleviate the problem of sparse rewards. By adjusting weights in stages, RL agents can remain robust in high-dimensional and multi-modal environments [10]; At the same time, the action space has expanded from simply adjusting F/CR to mutation strategy selection and even when to trigger L-BFGS. Notably, Tan et al. [11] proposed a differential evolution framework with hybrid parameters and mutation strategies based on reinforcement learning, which employs a hybrid policy network to simultaneously control mutation strategies and control parameters, demonstrating the effectiveness of continuous-continuous action space in DE optimization. Ding et al. [12] took the lead in implementing joint adaptive mutation operators and parameters within the DE framework using distributed proximal policy optimization. Yang et al. [13] further incorporated the timing of L-BFGS calls into RL decision-making, providing a scalable architecture template; In terms of multi-agent collaboration, Long et al. [14] systematically reviewed the progress of population level deep reinforcement learning, and Li Yao’s co evolutionary particle swarm framework [15] also showed that sub population information exchange can significantly improve high-dimensional exploration efficiency. Its divide and conquer approach lays a theoretical and empirical foundation for the future evolution of RL-DE towards multi-agent RL-DE. Furthermore, recent comprehensive surveys have systematically established the theoretical frameworks and research paradigms for reinforcement learning-assisted evolutionary algorithms (RL-EA), outlining the integration mechanisms between learning and optimization from both algorithmic design and application perspectives [16].

To address the sensitivity of differential evolution algorithm to parameters in complex optimization tasks, researchers have proposed various adaptive mechanisms. Specifically, JADE [17] demonstrates better convergence performance on various test functions by maintaining external archive and adaptively updating F and CR based on successful historical parameters; SaDE [18] learns mutation strategy selection and adaptive probability distributions of F and CR in real-time during the evolution process, forming an adaptive paradigm based on success history; SHADE [19] has become the basic template for high-performance DE variants by continuously archiving F and CR values and updating them using Lehmer mean; ILSHADE [20] introduces linear population size reduction (LPSR) and stricter external archive maintenance on this basis; JSO [21] further adopts a two-stage mutation pool and improved archive utilization, which is widely recognized as the strongest pure DE algorithm currently available and often serves as a benchmark for performance cap; MPEDE [22] divides the population into three subpopulations and configures different mutation strategies, while dynamically adjusting resources according to contribution, which can horizontally compare the simplicity and efficiency of RL-DE’s single population six dimensional continuous control.

Before the widespread application of reinforcement learning, researchers also attempted to dynamically adjust parameters through fuzzy logic, coding evolution, or random perturbations in order to improve algorithm robustness without the need for human intervention. Compared to fixed value schemes, FADE [3] uses fuzzy logic controllers to adjust F and CR online, making parameter changes more in line with population diversity fluctuations; JDE [23] encodes F and CR into individual genes and evolves synchronously with decision variables to achieve self optimizing parameter updates; RADE samples F through a simple random distribution, providing persistent perturbations without the need for additional storage; The dynamic population DE gradually reduces NP based on convergence progress, while maintaining exploration ability and reducing later computational costs. In addition, Brest et al. [23] systematically studied the impact of control parameter adaptivity on DE performance and verified the effectiveness of parameter adaptivity mechanisms on various numerical benchmark problems; Boggs and Byrd [24] proposed an adaptive finite memory BFGS algorithm from the perspective of quasi-Newton method, providing theoretical basis and algorithm foundation for the subsequent fusion of L-BFGS and DE.

Although significant progress has been made in parameter adaptation in the above research, its limitations are still evident: on the one hand, mainstream methods such as JADE, SaDE, SHADE generally rely on fixed mathematical formulas or statistical models to update F and CR, making it difficult to capture complex nonlinear dynamics in real-time during the optimization process; On the other hand, although early improvements such as FADE, jDE, RADE introduced ideas such as fuzzy logic, coding evolution, or random perturbations, the adjustment policy still belongs to the heuristic category, lacks learning mechanisms, and cannot dynamically optimize decisions based on historical experience, resulting in being easily trapped in inefficient parameter regions in complex or multimodal problems, making it difficult to achieve true intelligent control.

Based on the limitations of the above research, this article proposes RL-DE Algorithm, through the collaboration of reinforcement learning+quasi-Newton dual engines, achieves a leap in parameter adjustment from preset to self-learning. Its core contributions are as follows:

(1) Propose PG-Tuner: a policy gradient based reinforcement learning parameter controller that incorporates six dynamic parameters of differential evolution (DE) – mutation factor F, crossover probability CR, pBest selection ratio p, weight scaling factor Fw, archive disturbance intensity archF, archive usage probability archP - into a unified continuous action space, and models it as a Markov decision process through neural networks. It is worth noting that PG-Tuner focuses on adaptive adjustment of these dynamic parameters, while population size NP is pre specified by the user as a static hyperparameter based on the complexity of the problem. The controller uses the policy gradient method to update the policy online, which can synchronously adjust the six dimensional parameters during operation and gradually release the coupling potential between parameters; No need to rely on manual presets or fixed rules, allowing parameters to self adjust with the search process, providing support for the transition from global coarse exploration to local fine development.
(2) Propose a Two-Stage Mutation Switching mechanism: Design a dual-mode mutation strategy, using a globally exploratory strategy in the early stage (first 50% iterations) and switching to a locally exploratory strategy in the later stage. The switching threshold is based on a fixed iteration progress setting, which preserves the adaptive ability of the policy while avoiding the introduction of additional RL training overhead. It forms orthogonal complementarity with six parameter adjustment and constructs a parameter strategy dual layer collaborative framework, providing a feasible new idea for improving the search efficiency and convergence stability of complex multimodal problems.
(3) Propose a local refinement module LB-Refiner based on the L-BFGS algorithm: This module performs local refinement calculations with a fixed probability in the last 10% iterations of the DE run, quickly polishing the optimal or elite individuals. After the global search has locked in high-quality areas, it improves the accuracy of the solution at low cost and strengthens the algorithm’s local search ability.

The rest of this article is arranged as follows: Chapter 2 introduces the theoretical background and related work of differential evolution algorithm, policy gradient method, and L-BFGS algorithm; Chapter 3 elaborates on the proposed RL-DE algorithm, including the design and implementation of the PG-Tuner parameter controller, two-stage mutation strategy switching mechanism, and LB-Refiner local refinement module; Chapter 4 conducts a comprehensive experimental evaluation using the CEC 2014/2017 benchmark test function, and compares its performance, ablation experiments, and hyperparameter sensitivity analysis with multiple representative algorithms; Chapter 5 applies RL-DE to practical problems in flexible job shop scheduling and verifies its effectiveness in high-dimensional optimization scenarios; Chapter 6 summarizes the research findings and explores the limitations of the current framework and future research directions.

## 2. Theoretical background and related work

### 2.1. Differential evolution (DE) algorithm

Differential Evolution (DE) evolves populations through iterative execution of mutation, crossover, and selection operations [25].

(1) Mutation operation

For each target vector $\overset{\text{t}}{\underset{\text{i}}{\text{x}}}$ (i = 1,2,..., NP) in the population, perform mutation to generate a mutation vector v_i_ according to the following strategy:


$$\begin{array}{c} \overset{\text{t}}{\underset{\text{i}}{\text{v}}}=\overset{\text{t}}{\underset{\text{r1}}{\text{x}}}+\text{F}\cdot \left(\overset{\text{t}}{\underset{\text{r2}}{\text{x}}}-\overset{\text{t}}{\underset{\text{r3}}{\text{x}}}\right) \end{array}$$ (1)

Among them, $\overset{\text{t}}{\underset{\text{r1}}{\text{x}}},\overset{\text{t}}{\underset{\text{r2}}{\text{x}}},\overset{\text{t}}{\underset{\text{r3}}{\text{x}}}\in \left\{{\text{x}}_{\text{1}},\ldots {\text{x}}_{\text{NP}}\right\}$are three random individuals that are different from each other and have a different index i from the target vector; F∈(0, 1] is the scaling factor; NP is the population size.

(2) Cross operation

Perform binomial crossover on the target vector $\overset{\text{t}}{\underset{\text{i}}{\text{x}}}$ and the variation vector $\overset{\text{t}}{\underset{\text{i}}{\text{v}}}$ to generate the experimental vector $\overset{\text{t}}{\underset{\text{i}}{\text{u}}}$:


$$\begin{array}{c} \overset{\text{t}}{\underset{\text{i,j}}{\text{u}}}=\left\{ \begin{array}{c} @l@\overset{\text{t}}{\underset{\text{i,j}}{\text{v}}}\text{\textbackslash{}hspace\{0.17em }ifrand{\}}_{\text{d}}\left(0,1\right)\leq \text{CR\textbackslash{}hspace\{0.17em}\}or\text{\textbackslash{}hspace\{0.17em}\}j={\text{j}}_{\text{rand}}\\ \overset{\text{t}}{\underset{\text{i,j}}{\text{x}}}\text{ \textbackslash{}hspace\{0.17em}otherwise\} \end{array}\right. \end{array}$$ (2)

Among them, j∈{1,2,\ldots,D} is the dimension index; ${\text{rand}}_{\text{j}}\text{\textasciitilde{}U(0},\text{1)}$ is a uniformly distributed random number; CR∈[0,1] is the cross probability; j_rand_ is a dimension index randomly selected from {1,2,\ldots,D}.

(3) Select operation

Adopting a greedy selection strategy to retain better individuals:


$$\begin{array}{c} \overset{\text{t}+\text{1}}{\underset{\text{i}}{\text{x}}}=\left\{ \begin{array}{c} @l@\overset{\text{t}}{\underset{\text{i}}{\text{u}}}\text{\textbackslash{}hspace\{0.17em}\}if\text{ \textbackslash{}hspace\{0.17em}\}f\left(\overset{\text{t}}{\underset{\text{i}}{\text{u}}}\right)\leq \text{f}\left(\overset{\text{t}}{\underset{\text{i}}{\text{x}}}\right)\\ \overset{\text{t}}{\underset{\text{i}}{\text{x}}}\text{ \textbackslash{}hspace\{0.17em}otherwise\} \end{array}\right. \end{array}$$ (3)

Among them, f(.) is the objective function; $\overset{\text{t}+\text{1}}{\underset{\text{i}}{\text{x}}}$ is a new individual of the t + 1 generation. The above iterative process drives the population to move towards a more optimal region.

### 2.2. Policy gradient method

#### 2.2.1. Markov decision process.

Markov Decision Process (MDP) is a standard mathematical framework for describing sequential decision problems [26], consisting of five tuples (S, A, P, R, γ): S is the state space that summarizes the current observable information of the system; A is the action space, defining the control variables that decision-makers can choose from; P provides the transition probability of state action pairs to the next state, characterizing the uncertainty of the environment; R provides real-time profit signals for evaluating the quality of actions; γ∈[0,1] is a discount factor used to balance immediate and future rewards.

The state transition probability formula of Markov decision process is as follows:


$$\begin{array}{c} \text{P}\left({\text{s}}_{\text{t}+\text{1}}|{\text{s}}_{\text{t}},{\text{a}}_{\text{t}}\right)\;=\;\text{Pr}\left({\text{s}}_{\text{t}+\text{1}}|{\text{s}}_{\text{t}},{\text{a}}_{\text{t}}\right) \end{array}$$ (4)

Among them, s_t_ ∈ S is the state of step t; a_t_∈A is the action performed at step t; s_t+1_ is the next state after the transition.

The probability of the reward function formula is as follows:


$$\begin{array}{c} \text{R}\left({\text{s}}_{\text{t}},{\text{a}}_{\text{t}}\right)=\text{E}\left[{\text{r}}_{\text{t}}|{\text{s}}_{\text{t}},{\text{a}}_{\text{t}}\right] \end{array}$$ (5)

Among them, r_t_ ∈ R is the immediate reward obtained in step t; E [.] represents the expected operator.

The goal is to find the optimal policy π^*^(a | s) to maximize the expected cumulative return:


$$\begin{array}{c} \text{J(}\pi \text{)}={\text{E}}_{\pi }\left[\sum_{\text{t}\;=\;\text{0}}^{\infty }\gamma^{\text{t}}{\text{r}}_{\text{t}}\right] \end{array}$$ (6)

Among them, π(a | s) is the policy function, representing the probability of selecting action a in state s; E_π_[.] represents the expectation under policy π; This objective function takes into account both immediate rewards and long-term discount rewards [26].

#### 2.2.2. Gradient of continuous control policy.

The policy gradient method solves the continuous control MDP [27] by directly optimizing the parameterized policy π_θ_(a | s), where θ ∈ R^d^ is the policy network parameter. This method uses the policy gradient theorem to calculate the gradient of the objective function J(θ) with respect to the policy parameters:


$$\begin{array}{c} \nabla_{\theta }\text{\textbackslash{}hspace\{0.17em}J(\}\theta \text{)}={\text{E}}_{\pi }\left[\nabla_{\theta }log\pi_{\theta }\left(\text{a}|\text{s}\right)\cdot {\text{Q}}^{\pi }\left(\text{s},\text{a}\right)\right] \end{array}$$ (7)

Among them, ${\text{Q}}^{\pi }\left(\text{s},\text{a}\right)={\text{E}}_{\pi }\left[\sum_{\text{k}=\text{0}}^{\infty }\gamma^{\text{k}}{\text{r}}_{\text{t}+\text{k}}|{\text{s}}_{\text{t}}=\text{s},{\text{a}}_{\text{t}}=\text{a}\right]\;$is the state action value function, which represents the expected cumulative return following policy π after executing action a in state s; $\nabla_{\theta }{log\pi }_{\theta }\left(\text{a}|\text{s}\right)$ is the logarithmic policy gradient, which guides the direction of parameter updates.

To reduce the variance of gradient estimation, the dominance function A^π^(s, a) is used instead of Q^π^(s, a) in practice:


$$\begin{array}{c} {\text{A}}^{\pi }{\text{(s,a)\textasciitilde{}=\textasciitilde{}Q}}^{\pi }{\text{(s,a)-V}}^{\pi }\text{(s)} \end{array}$$ (8)

Among them, ${\text{V}}^{\pi }\text{(s)}={\text{E}}_{\pi }\left[{\text{Q}}^{\pi }\left(\text{s},\text{a}\right)\right]$ is the state value function, representing the expected return of state s. The advantage function highlights the superiority or inferiority of action a relative to the average level by subtracting the baseline V^π^(s).

Based on Monte Carlo sampling, the policy gradient can be approximated as:


$$\begin{array}{c} \nabla_{\theta }J(\theta )\approx \frac{\text{1}}{\text{N}}\sum_{\text{i}=\text{1}}^{\text{N}}\sum_{\text{t}=\text{0}}^{\text{T}}\nabla_{\theta }log\pi_{\theta }\left({\text{a}}_{\text{i},\text{t}}|{\text{s}}_{\text{i},\text{t}}\right)\cdot {\text{R}}_{\text{i},\text{t}} \end{array}$$ (9)

Among them, N is the number of sampling trajectories; T is the maximum number of steps per trajectory; ${\text{R}}_{\text{i},\text{t}}=\sum_{{\text{t}}^{\prime }=\text{t}}^{\text{T}}\gamma^{{\text{t}}^{\prime }-\text{t}}{\text{r}}_{\text{i},{\text{t}}^{\prime }}$ is the discount return for the i-th trajectory at step t. This estimation enables the policy network to learn optimization from experience and achieve adaptive fine control of DE parameters [28].

### 2.3. L-BFGS algorithm

L-BFGS(Limited-memory Broyden-Fletcher-Goldfarb-Shanno) Algorithm [29] is an efficient quasi-Newton optimization method specifically designed to solve large-scale unconstrained optimization problems. It is an improved version of the BFGS algorithm that reduces memory usage, allowing it to handle optimization problems with a large number of variables.

The core idea of the L-BFGS algorithm is to accelerate the convergence speed of gradient descent by approximating the inverse of the Hessian matrix. Unlike the traditional BFGS algorithm, the L-BFGS algorithm only stores the gradient information and position change information of the last few iterations, rather than storing the complete inverse of the Hessian matrix. This greatly reduces memory requirements, making it suitable for large-scale problems.

The iterative core of L-BFGS lies in efficiently calculating the search direction and updating the approximation matrix. Each iteration mainly consists of three interrelated steps:

(1) Calculation of search direction

In the kth iteration, the algorithm first approximates the search direction d_k_ based on the gradient g_k_ of the current point and the inverse of the Hessian matrix H_k_, and obtains the search direction dk through simple multiplication:


$$\begin{array}{c} {\text{d}}_{\text{k}}=-{\text{H}}_{\text{k}}{\text{g}}_{\text{k}} \end{array}$$ (10)

Among them, ${\text{g}}_{\text{k}}=\nabla {\text{f(x}}_{\text{k}}\text{)}$ is the gradient at the current point x_k_, and H_k_ is an approximation of the inverse of the Hessian matrix.

(2) Update of position and gradient

After determining the search direction, the algorithm determines the step size α_k_ through line search (satisfying the Wolfe condition) and updates the position and gradient:


$$\begin{array}{c} \text{Location update }{\text{x}}_{\text{k}+\text{1}}{=\text{x}}_{\text{k}}{+\alpha }_{\text{k}}{\text{d}}_{\text{k}} \end{array}$$ (11)

$$\begin{array}{c} \text{Gradient update }{\text{g}}_{\text{k}+\text{1}}=\nabla {\text{f}(\text{x}}_{\text{k}+\text{1}}) \end{array}$$ (12)

(3) Implicit update of approximate matrix

L-BFGS does not directly store H_k_, but implicitly maintains matrix information by storing the last m pairs of {s_i_, y_i_} vectors (i = k-m,..., k-1). among which


$$\begin{array}{c} \text{Position variation }{\text{s}}_{\text{k}}{=\text{x}}_{\text{k}+\text{1}}-{\text{x}}_{\text{k}} \end{array}$$ (13)

$$\begin{array}{c} \text{Gradient variation }{\text{y}}_{\text{k}}{=\text{g}}_{\text{k}+\text{1}}-{\text{g}}_{\text{k}} \end{array}$$ (14)

These vectors capture key information about the curvature of the objective function. Based on them, BFGS updated the approximate formula for the inverse of the Heisenberg matrix:


$$\begin{array}{c} {\text{H}}_{\text{k}+\text{1}}=\left(\text{I}-\rho_{\text{k}}{\text{s}}_{\text{k}}\overset{\text{T}}{\underset{\text{k}}{\text{y}}}\right){\text{H}}_{\text{k}}\left(\text{I}-\rho_{\text{k}}{\text{y}}_{\text{k}}\overset{\text{T}}{\underset{\text{k}}{\text{s}}}\right)+\rho_{\text{k}}{\text{s}}_{\text{k}}\overset{\text{T}}{\underset{\text{k}}{\text{s}}} \end{array}$$ (15)

Among them, the scaling factor $\rho_{\text{k}}=\frac{\text{1}}{\overset{\text{T}}{\underset{\text{k}}{\text{y}}}{\text{s}}_{\text{k}}}$ is used to ensure scale invariance.

To avoid explicit calculation and storage of H_k_, L-BFGS uses a double loop recursive algorithm to directly calculate H_k_g_k_, with the following process:

Forward loop: Starting from the most recent iteration, traverse in reverse, calculate intermediate variables $\alpha_{\text{i}}=\rho_{\text{i}}\overset{\text{T}}{\underset{\text{i}}{\text{s}}}\text{q}$ and update $\text{q}=\text{q}-\alpha_{\text{i}}{\text{y}}_{\text{i}}$, gradually stripping away the influence of historical curvature information.

Scaling steps: Use the initial scale $\gamma =\frac{\overset{\text{T}}{\underset{\text{k}-\text{1}}{\text{s}}}{\text{y}}_{\text{k}-\text{1}}}{\overset{\text{T}}{\underset{\text{k}-\text{1}}{\text{y}}}{\text{y}}_{\text{k}-\text{1}}}$ to scale q and obtain the initial search direction r.

Reverse loop: Starting from the farthest iteration, traverse forward, calculate $\beta =\rho_{\text{i}}\overset{\text{T}}{\underset{\text{i}}{\text{y}}}\text{r}$ and update $\text{r}=\text{r}+\left(\alpha_{\text{i}}-\beta \right){\text{s}}_{\text{i}}$, re inject historical curvature information, and finally obtain the accurate search direction ${\text{d}}_{\text{k}}=-\text{r}$, which will be directly used for position update (see formula 11).

## 3. Proposed RL-DE algorithm

**Fig 1:**
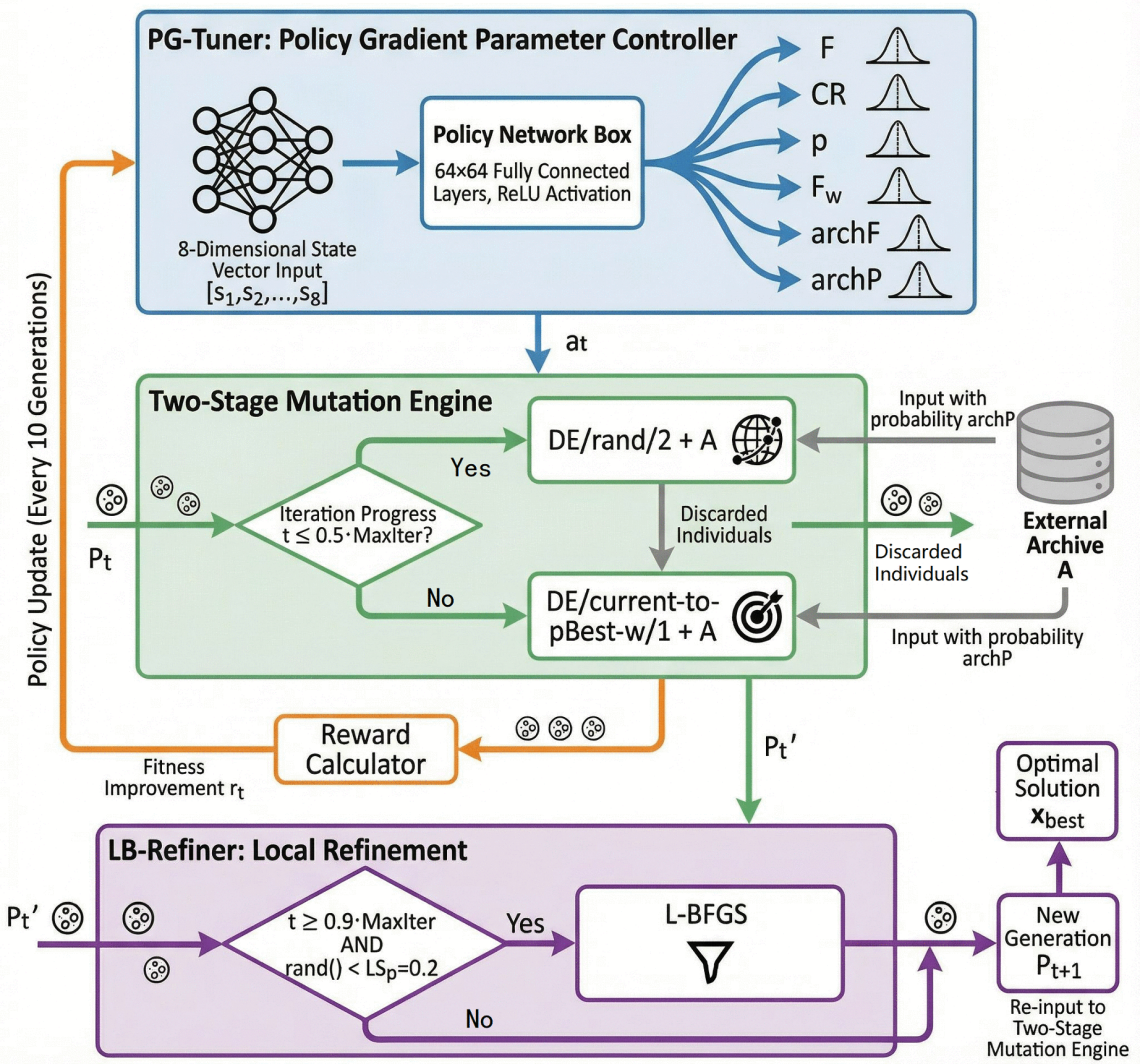
RL-DE framework schematic diagram.

The RL-DE algorithm achieves a paradigm shift in differential evolution from heuristic control to intelligent learning through a three-layer collaborative framework of policy parameter refinement. The core idea is: the two-stage mutation strategy provides the basic search skeleton → PG-Tuner dynamically adjusts the six dimensional parameters → LB-Refiner locally refines the solution accuracy, and the three form a closed-loop feedback. The complete architecture and information flow of the three-layer collaborative framework are shown in Fig 1. This chapter first elaborates on the external archive and strategy switching of the two-stage mutation mechanism, then describes the neural network embedding and MDP modeling process of PG-Tuner, and finally explains the local refinement policy and overall algorithm flow of L-BFGS.

### 3.1. Dual stage mutation strategy switching mechanism

RL-DE balances global exploration and local development through a two-stage mutation strategy that switches at a fixed iteration threshold, with all dynamic parameters adaptively generated by PG-Tuner (Section 3.3). Together with the external archive, these form the search backbone (Fig 1). In the early stage, the DE/rand/2 + A strategy is employed, utilizing double difference vectors and archive perturbations to generate high-intensity randomness for exploring complex multimodal spaces. The mathematical expression for this strategy is:


$$\begin{array}{c} \begin{array}{c} \text{if\textbackslash{}hspace\{0.17em}t\}\leq 0.5\text{T:}\\ \overset{\text{(t)}}{\underset{\text{i}}{\text{V}}}=\left\{ \begin{array}{c} @l@\overset{\text{(t)}}{\underset{\text{r1}}{\text{X}}}+\text{F}\cdot \left(\overset{\text{(t)}}{\underset{\text{r2}}{\text{X}}}-\overset{\text{(t)}}{\underset{\text{r3}}{\text{X}}}\right)+\text{F}\cdot \left(\overset{\text{(t)}}{\underset{\text{r4}}{\text{X}}}-\overset{\text{(t)}}{\underset{\text{r5}}{\text{X}}}\right)\text{+archF}\cdot \left(\overset{\text{(t)}}{\underset{\text{r6}}{\text{X}}}-{\text{X}}_{\text{a}}\right)\text{if\textbackslash{}hspace\{0.17em}rand\}\leq \text{archP}\\ \overset{\text{(t)}}{\underset{\text{r1}}{\text{X}}}+\text{F}\cdot \left(\overset{\text{(t)}}{\underset{\text{r2}}{\text{X}}}\overset{\text{(t)}}{\underset{\text{r3}}{\text{-X}}}\right)+\text{F}\cdot \left(\overset{\text{(t)}}{\underset{\text{r4}}{\text{X}}}-\overset{\text{(t)}}{\underset{\text{r5}}{\text{X}}}\right)\text{\textbackslash{}hspace\{0.17em}\;\;otherwise\} \end{array}\right. \end{array} \end{array}$$ (16)

Among them, t is the current iteration number and T is the total number of iterations; $\overset{\text{(t)}}{\underset{\text{r1}}{\text{X}}}$, $\overset{\text{(t)}}{\underset{\text{r2}}{\text{X}}}$, $\overset{\text{(t)}}{\underset{\text{r3}}{\text{X}}}$, $\overset{\text{(t)}}{\underset{\text{r4}}{\text{X}}}$, $\overset{\text{(t)}}{\underset{\text{r5}}{\text{X}}}$, and $\overset{\text{(t)}}{\underset{\text{r6}}{\text{X}}}$ are six distinct individuals randomly selected from the current population P^(t)^ at generation t, all different from the target individual $\overset{\text{(t)}}{\underset{\text{i}}{\text{X}}}$; the two difference vector pairs ($\overset{\text{(t)}}{\underset{\text{r2}}{\text{X}}}$-$\overset{\text{(t)}}{\underset{\text{r3}}{\text{X}}}$) and ($\overset{\text{(t)}}{\underset{\text{r4}}{\text{X}}}$-$\overset{\text{(t)}}{\underset{\text{r5}}{\text{X}}}$) are mutually exclusive (non-overlapping indices), providing anisotropic perturbations; archF∈[0, 1] is the archive disturbance intensity (scaling factor for archive-based perturbation), archP∈[0, 1] is the archive usage probability (probability of incorporating archive individuals), X_a_ is an individual randomly selected from the external archive set A, and rand~U(0, 1) is a uniform random number in [0, 1]; F is the mutation scaling factor (differential weight).

As the iteration enters the later stage, the algorithm switches to the DE/current to pBest-w/1 + A strategy, utilizing the current optimal solution information to achieve targeted fine development and accelerate the convergence of high-quality areas. The expression is:


$$\begin{array}{c} \begin{array}{c} \text{if\textbackslash{}hspace\{0.17em}t\}>\text{0.5T:}\\ \overset{\text{(t)}}{\underset{\text{i}}{\text{V}}}=\left\{ \begin{array}{c} @l@\overset{\text{(t)}}{\underset{\text{i}}{\text{X}}}+\text{Fw}\cdot \left(\overset{\text{(t)}}{\underset{{\text{pBest}}_{\text{i}}}{\text{X}}}-\overset{\text{(t)}}{\underset{\text{i}}{\text{X}}}\right)+\text{F}\cdot \left(\overset{\text{(t)}}{\underset{\text{r1}}{\text{X}}}-\overset{\text{(t)}}{\underset{\text{r2}}{\text{X}}}\right)+\text{archF}\cdot \left(\overset{\text{(t)}}{\underset{{\text{pBest}}_{\text{j}}}{\text{X}}}-\overset{\text{(t)}}{\underset{\text{a}}{\text{X}}}\right)\text{\textbackslash{}hspace\{0.17em}if\;rand\}\leq \text{archP}\\ \overset{\text{(t)}}{\underset{\text{i}}{\text{X}}}+\text{Fw}\cdot \left(\overset{\text{(t)}}{\underset{{\text{pBest}}_{\text{i}}}{\text{X}}}-\overset{\text{(t)}}{\underset{\text{i}}{\text{X}}}\right)+\text{F}\cdot \left(\overset{\text{(t)}}{\underset{\text{r1}}{\text{X}}}-\overset{\text{(t)}}{\underset{\text{r2}}{\text{X}}}\right)\text{\textbackslash{}hspace\{0.17em}\;otherwise\} \end{array}\right. \end{array} \end{array}$$ (17)

The implementation details are as follows:

(1) pBest selection: The p∈[0,1] value output by PG-Tuner is the pBest selection ratio, which is used to determine the number of elite individuals ${\text{p}}_{\text{cnt}}\;=\text{max(1},\text{p}\cdot \text{NP)}$. ${\text{X}}_{{\text{pBest}}_{\text{i}}}$ is randomly selected from the top p_cnt_ optimal individuals with replacement;
(2) Weighted guidance: Fw is the weight scaling factor output by PG-Tuner, specifically used to control the step strength of guiding the current individual to pBest;
(3) Archive perturbation: Using probability archP to select ${\text{X}}_{{\text{pBest}}_{\text{j}}}$ and external archive individuals X_a_ from the pBest set to form a secondary perturbation, maintaining moderate diversity in the later stages of development.

The two-stage mutation strategy shares the same external archive A and randomly introduces archive individuals using probability archP to maintain diversity. The external archive set A enhances population diversity and guides algorithms to avoid invalid search areas through the mechanism of eliminating individuals for reuse. Its construction and maintenance follow the following rules:

(1) Initialization and Capacity Management
When the algorithm starts, the external archive is initialized to an empty list structure, with its maximum capacity set to the problem dimension D, that is, |A|_max_ = D. This list structure supports dynamic append and random access to ensure access efficiency.
(2) Eliminating individual storage mechanisms
In each generation selection stage, when the experimental individual $\overset{\text{t}}{\underset{\text{i}}{\text{U}}}$ successfully replaces the target individual $\overset{\text{t}}{\underset{\text{i}}{\text{X}}}$, the eliminated old individual is stored in the archive with an exponential decay probability. The probability p_save_(t) dynamically adjusts with the number of iterations t:

$$\begin{array}{c} {\text{p}}_{\text{save}}\text{(t)}={\text{p}}_{\text{min}}+\left({\text{p}}_{\text{max}}-{\text{p}}_{\text{min}}\right)\cdot \overset{\text{t}}{\underset{\text{arch}}{\gamma }} \end{array}$$ (18)

Among them, p_max_ = 1.0 is the initial storage probability, p_min_ = 0.01 is the last generation storage probability, and the decay coefficient γ_arch_ = 0.98 controls the rate of probability decrease.
(3) Archive overflow handling strategy
When the file reaches the capacity limit, the first in first out strategy is no longer used, but the maximum and minimum distance replacement is performed to maintain population spatial diversity:

Let the set of individuals in the current file be $\overset{\text{D}}{\underset{\text{j}=\text{1}}{\left\{\overset{\text{j}}{\underset{\text{a}}{\text{X}}}\right\}}}$, and the newly eliminated individual be X_new_. Firstly, calculate the minimum distance from the new individual to all individuals in the archive:


$$\begin{array}{c} \overset{\text{new}}{\underset{\text{min}}{\text{d}}}=\underset{\text{j}}{\text{min}}{\|{\text{X}}_{\text{new}}-\overset{\text{j}}{\underset{\text{a}}{\text{X}}}\|}_{\text{2}} \end{array}$$ (19)

Calculate the minimum distance from each individual $\overset{j}{\underset{a}{\text{X}}}$ in the archive to the remaining individuals:


$$\begin{array}{c} \overset{\text{j}}{\underset{\text{min}}{\text{d}}}=\underset{\text{k}\neq \text{j}}{\text{min}}{\|\overset{\text{j}}{\underset{\text{a}}{\text{X}}}-\overset{\text{k}}{\underset{\text{a}}{\text{X}}}\|}_{\text{2}} \end{array}$$ (20)

Identify the nearest pair of individual indices $\left(\overset{\text{*}}{\underset{\text{1}}{\text{j}}},\overset{\text{*}}{\underset{\text{2}}{\text{j}}}\right)={\text{argmin}}_{\text{j},\text{k}}{\|\overset{\text{j}}{\underset{\text{a}}{\text{X}}}-\overset{\text{k}}{\underset{\text{a}}{\text{X}}}\|}_{\text{2}}$ in the archive and randomly select one as a candidate replacement index ${\text{j}}_{\text{victim}}\in \left\{\overset{\text{*}}{\underset{\text{1}}{\text{j}}},\overset{\text{*}}{\underset{\text{2}}{\text{j}}}\right\}$. If $\overset{\text{new}}{\underset{\text{min}}{\text{d}}}>\overset{\text{victim}}{\underset{\text{min}}{\text{d}}}$ is satisfied, indicating that the new individual is more dispersed in spatial distribution, then replace $\overset{\text{victim}}{\underset{\text{a}}{\text{X}}}$ with X_new_; Otherwise, abandon storage. This strategy prioritizes preserving historical information with a more dispersed spatial distribution while controlling the size of archives, improving the efficiency of archive utilization.

### 3.2. Local refinement (LB-Refiner) module

PG-Tuner achieves efficient global search of differential evolution (DE) through dynamic parameter generation, but after locking high-quality regions in the global search, it is difficult to further improve the accuracy of the solution solely through parameter adjustment. To this end, the LB-Refiner module – a heuristic local refinement component based on the L-BFGS algorithm – is introduced to perform low-cost refinement in local areas, forming a closed-loop optimization of parameter control+local refinement. As shown in Fig 1, LB-Refiner intervenes as an independent module in the final stage of optimization, forming a three-layer collaborative framework with PG-Tuner and two-stage mutation.

The specific implementation of the LB-Refiner module in the RL-DE algorithm has been described in detail in Algorithm 3 (lines 23–28). When the iteration reaches the last 10% (t ≥ 0.9 T), each generation triggers LB-Refiner with a probability of LS_P = 0.2. If triggered, randomly select an individual from the current best or top 5% elite individual as the L-BFGS initial point, and use the L-BFGS algorithm to locally optimize the selected initial point (see Algorithm 1 for the specific process). After the optimization is completed, if the new solution is better than the population optimum, the external archive will be directly replaced and synchronously updated, and only one individual will be refined per generation to maintain population diversity. This policy is expected to improve convergence speed and resolution accuracy in local stages without the need for additional manual intervention.

**Algorithm 1** **LB-Refiner local refinement process**

Input:

 • Initial point x_0_ (currently the best or elite individual in the population)

 • f(x) (fitness function)

 • g(x) (The gradient vector of f(x))

 • MaxIter (maximum number of L-BFGS iterations, maxIter=30)

 • ε_g_ (gradient norm convergence threshold, ε_g_=1e-5)

Output:

 • Refine the solution x*

1: x ← x₀

2: g ← g(x)

3: If ||g|| ≤ ε_g_ then return x # The first-order optimum has been satisfied, and there is no need for local optimization

4: for k = 1 to maxIter do

5: d ← - H_k_ g # Calculate the search direction recursively in a double loop according to formula 10

6: α ← LineSearchWolfe(f, g, x, d) # satisfies formulas 11 and 12

7: x ← x + αd

8: g ← g(x) # Update gradient

9: Update memory for {s_k_, y_k_} # according to formula (13) (14)

10: If ||g|| ≤ ε_g_ then return x # Convergence judgment

11:end for

12:return x

### 3.3. Policy gradient tuner (PG-Tuner)

**Fig 2:**
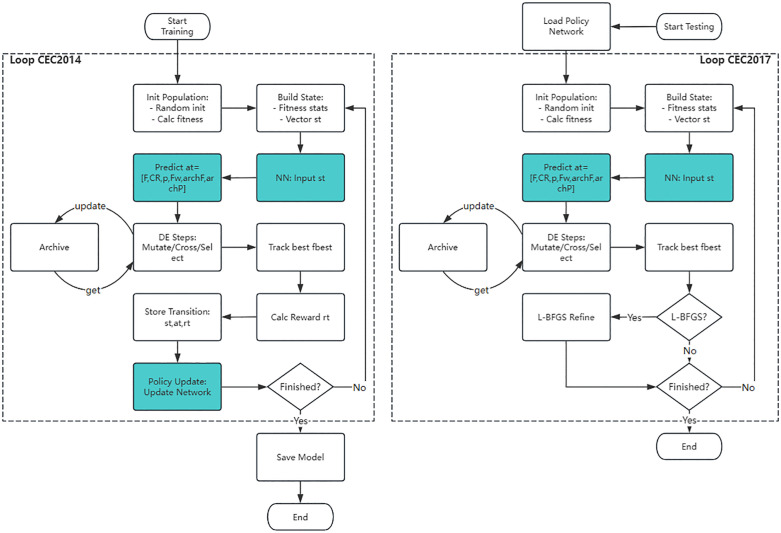
Schematic diagram of embedding PG-Tuner neural network into DE framework.

PG-Tuner is the intelligent decision-making core of RL-DE, which is essentially a fully connected neural network that learns the optimal control policy for six key parameters (F, CR, p, Fw, archF, archP) online through policy gradient method. As shown in Fig 1, the PG-Tuner takes the current population statistical characteristics as input and outputs a six dimensional parameter distribution, directly driving the DE engine’s mutation and crossover operations, forming a closed loop of state observation → policy decision-making → environmental feedback. Unlike traditional adaptive methods that rely on fixed statistical rules, PG-Tuner models parameter control as a continuous Markov decision process, achieving a transition from rule driven to data-driven. The core embedding mechanism is shown in Fig 2.

Unlike traditional adaptive methods that rely on fixed statistical rules, PG-Tuner models parameter control directly as a continuous MDP, achieving a transition from rule driven to data-driven; To achieve this, it is necessary to first embed the neural network into the DE framework and then provide a complete formal description of MDP.

In the standard DE framework, parameters F, CR, etc. are usually generated by fixed rules or random perturbations, and this decoupling method ignores the nonlinear coupling effects between parameters. PG-Tuner embeds a parameterized neural network π_θ_ to map the state observation s_t_ to a Gaussian distribution N(μ, σ^2^) of six parameters, achieving collaborative optimization among parameters. In each generation t, PG-Tuner takes the current population statistical characteristics as input and outputs the distribution parameters of six dimensional parameters a_t_=[F, CR, p, Fw, archF, archP]; Obtain specific action values through reparameterization sampling, and guide DE mutation and crossover operations after truncation; After executing the operation, the environment (DE engine) returns a new state s_t+1_ and a reward r_t_, forming a closed loop. This embedding method deeply integrates parameter control with DE evolution, making the policy network an inseparable decision brain of the algorithm.

#### 3.3.1. MDP modeling.

Next, the DE parameter control problem will be formalized as a Markov decision process for direct optimization using the policy gradient method. To apply the policy gradient method, the DE parameter control problem needs to be formalized as a Markov decision process MDP=(S, A, P, R, γ). The specific construction of the quintuple is given in order below. First, the state space (S) is described.

The state vector s_t_ ∈ R^8^ is constructed from the population fitness distribution and search progress in each generation. Specifically, the state consists of seven normalized fitness statistics plus a logarithmic progress indicator, defined as follows:

(1) Logarithmic scaling: Performing a unified logarithmic scaling on population fitness to compress differences in magnitude.

$$\begin{array}{c} \overset{\text{log}}{\underset{\text{i}}{\text{f}}}=\text{sgn}\left({\text{f}}_{\text{i}}\right)\cdot \text{log}\left(\left|{\text{f}}_{\text{i}}\right|+\text{1e}-\text{8}\right)\text{,\textbackslash{}hspace\{0.17em}i\}=\text{1},...,\text{NP} \end{array}$$ (21)

Among them, sgn is the sign function; log represents the common logarithm base 10 (log_10_), the same throughout the text; To prevent it from being 0, an additional 1e-8 is added, the same throughout the text.
(2) Statistical extraction: Extract 7 statistical measures on logarithmic fitness:
  - f_min_: minimum fitness value
  - f_25%_: 25th percentile fitness (first quartile, Q1)
  - f_med_: median fitness (50th percentile, Q2)
  - f_75%_: 75th percentile fitness (third quartile, Q3)
  - f_max_: maximum fitness value
  - f_mean_: mean fitness value (average)
  - f_std_: standard deviation of fitness distribution

$$\begin{array}{c} \text{stats}=\left[{\text{logf}}_{\text{min}},{\text{logf}}_{\text{25\textbackslash{}\%}},{\text{logf}}_{\text{med}},{\text{logf}}_{\text{75\textbackslash{}\%}},{\text{logf}}_{\text{max}},{\text{logf}}_{\text{mean}},{\text{logf}}_{\text{std}}\right] \end{array}$$ (22)

(3) Relative normalization: Based on the median, normalize the six statistical measures other than the median to make the state values independent of the problem scale.

$$\begin{array}{c} {\tilde{\text{s}}}_{\text{i}}=\frac{{\text{stats}}_{\text{i}}-{\text{logf}}_{\text{med}}}{{\text{logf}}_{\text{max}}-{\text{logf}}_{\text{min}}+\text{1e-8}}\text{, i}\in \left\{\text{0},\text{1},\text{2},\text{3},\text{4},\text{5},\text{6}\right\} \end{array}$$ (23)

(4) Progress feature: Introducing search progress feature – taking the logarithm of the proportion of currently consumed generations to the total generations, reflecting the iteration progress.
(5) Final state: Assemble into an 8-dimensional vector s_t_ – 6 normalized statistics+median fixed to 0 + logarithmic progression:

$$\begin{array}{c} {\text{s}}_{\text{t}}={\left[{\tilde{\text{s}}}_{\text{min}},{\tilde{\text{s}}}_{\text{25\textbackslash{}\%}},\text{0},{\tilde{\text{s}}}_{\text{75\textbackslash{}\%}},{\tilde{\text{s}}}_{\text{max}},{\tilde{\text{s}}}_{\text{mean}}\text{,}{\tilde{\text{s}}}_{\text{std}},\text{log(t/T)}\right]}^{\text{T}} \end{array}$$ (24)

Among them, t/T represents the iteration progress, where t denotes the current iteration number and T denotes the total number of iterations.

Once the state is determined, the six dimensional continuous control variables are defined as a unified action vector at once. The action space is composed of six continuous parameter vectors:


$$\begin{array}{c} \text{A}=\left\{\left[\text{F},\text{CR},\text{p},\text{Fw},\text{archF},\text{archP}\right]|\text{F},\text{CR},\text{p},\text{Fw},\text{archF},\text{archP}\in \left[\text{0},\text{1}\right]\right\} \end{array}$$ (25)

Each component is controlled separately: F∈[0,1] is the variation factor, CR∈[0,1] is the crossover probability, p∈[0,1] is the pBest selection ratio, Fw∈[0,1] is the weight scaling factor, archF∈[0,1] is the archive disturbance intensity, and archP∈[0,1] is the archive usage probability. The policy network π_θ_ outputs the Gaussian distribution mean μ ∈ R^6^ of each parameter, and then obtains the valid action values through sampling and truncation.

Once the action is executed, the population enters the next generation according to DE dynamics, which implies a state transition. The state transition is implicitly defined by the dynamics of the DE algorithm:


$$\begin{array}{c} \text{P}\left({\text{s}}_{\text{t}+\text{1}}|{\text{s}}_{\text{t}},{\text{a}}_{\text{t}}\right)=\left\{ \begin{array}{c} @l@\text{1\textbackslash{}hspace\{0.17em}if\;s{\}}_{\text{t}+\text{1}}=\text{DE}\left({\text{s}}_{\text{t}},{\text{a}}_{\text{t}}\right)\\ \text{0\textbackslash{}hspace\{0.17em}otherwise\} \end{array}\right. \end{array}$$ (26)

Among them, DE(.) represents the new state generated after performing one generation mutation, crossover, and selection operations. The transfer is deterministic, completely determined by the current population and parameters.

To guide policy learning, it is also necessary to immediately return a concise and effective reward signal in each generation. To balance computational efficiency and policy learnability, a minimalist reward design is adopted here. Each generation only monitors three front-end indicators of population fitness distribution – optimal value, 10% percentile value, and 25% percentile value, compares them with the corresponding indicators of the previous generation for relative improvement, and then logarithmically scales them as immediate rewards. This signal reflects the overall progress of the population within a single generation, enabling the reinforcement learning agent to quickly and stably capture the instantaneous changes in optimized terrain.


$$\begin{array}{c} {\text{r}}_{\text{t}}=\text{log}\left(\frac{\overset{\left(\text{t}-\text{1}\right)}{\underset{\text{best}}{\text{f}}}-\overset{\text{(t)}}{\underset{\text{best}}{\text{f}}}}{\left|\overset{\text{(t-1)}}{\underset{\text{best}}{\text{f}}}\right|}+0.2\cdot \frac{\overset{\text{(t-1)}}{\underset{\text{10\textbackslash{}\%}}{\text{f}}}-\overset{\text{(t)}}{\underset{\text{10\textbackslash{}\%}}{\text{f}}}}{\left|\overset{\text{(t-1)}}{\underset{\text{10\textbackslash{}\%}}{\text{f}}}\right|}+\text{0.1}\cdot \frac{\overset{\text{(t-1)}}{\underset{\text{25\textbackslash{}\%}}{\text{f}}}-\overset{\text{(t)}}{\underset{\text{25\textbackslash{}\%}}{\text{f}}}}{\left|\overset{\text{(t-1)}}{\underset{\text{25\textbackslash{}\%}}{\text{f}}}\right|}\right) \end{array}$$ (27)

Among them, $\overset{\text{(t)}}{\underset{\text{best}}{\text{f}}}$, $\overset{\text{(t)}}{\underset{\text{10\textbackslash{}\%}}{\text{f}}}$, and $\overset{\text{(t)}}{\underset{\text{25\textbackslash{}\%}}{\text{f}}}$ represent the best fitness value, the 10th percentile, and the 25th percentile of the population at generation t, respectively; $\overset{\text{(t-1)}}{\underset{\text{best}}{\text{f}}}$, $\overset{\text{(t-1)}}{\underset{\text{10\textbackslash{}\%}}{\text{f}}}$, and $\overset{\text{(t-1)}}{\underset{\text{25\textbackslash{}\%}}{\text{f}}}$ denote the corresponding values at generation t-1 (previous generation); the superscripts (t) and (t-1) indicate the current and previous generation indices, respectively; log denotes the base-10 logarithm.

With instant rewards, the ultimate goal can be expressed as maximizing the expected discount cumulative return. The output action distribution of the policy network π_θ_ is π_θ_(a | s)=N(μ_θ_(s), σ^2^). The goal of PG-Tuner is to maximize the expected cumulative discount return from the initial state s_0_:


$$\begin{array}{c} \text{J(}\theta \text{)}={\text{E}}_{\text{r }\pi \theta }\left[\sum_{\text{t}=\text{0}}^{\text{T}}\gamma^{\text{t}}{\text{r}}_{\text{t}}|{\text{s}}_{\text{0}}\right] \end{array}$$ (28)

The trajectory $\tau =\left\{{\text{s}}_{\text{0}},{\text{a}}_{\text{0}},{\text{r}}_{\text{0}},...,{\text{s}}_{\text{T}},{\text{a}}_{\text{T}},{\text{r}}_{\text{T}}\right\}$ is generated by the interaction between the policy π_θ_ and the environment, with γ as the discount factor used to balance immediate and future rewards, making the agent more focused on long-term optimization performance. Here, γ = 0.9. Update the parameter θ through the policy gradient theorem.

#### 3.3.2. Policy network training and embedding.

With precise definitions of state, action, and reward, the training details of the policy network can be derived. The policy network is trained using the REINFORCE algorithm, which is a classic policy gradient method. The training process includes running the DE algorithm using the current policy to complete a complete scenario (performing a complete optimization run on the problem), recording the sequence of states, actions, and rewards, estimating the policy gradient at the end of the scenario, and updating the network weight θ to maximize the expected cumulative reward. To this end, follow the three steps of network structure gradient calculation training process in sequence.

**Fig 3:**
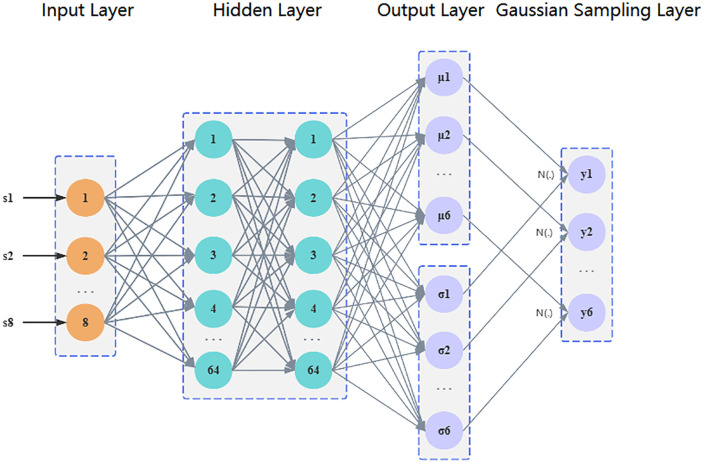
PG-Tuner policy network architecture diagram. Note: In Fig 3, (s1, s2,..., s8) are 8-dimensional state vectors, (y1, y2,... y6) are 6-dimensional control vectors, corresponding to (F, CR, p, Fw, archF, archP), and N(.) represents Gaussian sampling based on μ and σ, respectively.

Firstly, the structure of the policy gradient network is explained. The parameterized policy π_θ_ is implemented by a fully connected neural network, as shown in Fig 3. The network takes an 8-dimensional state vector s_t_ as input, and after two hidden layers (64 neurons per layer, ReLU activated), outputs a six dimensional Gaussian distribution mean vector μ_θ_(s_t_). The logarithmic standard deviation logσ is stored independently as a trainable parameter to ensure exploration stability.

Once the network structure is established, the direction of parameter updates can be derived based on the policy gradient theorem. According to the policy gradient theorem, the gradient of the objective function J(θ) with respect to the policy parameters is:


$$\begin{array}{c} \nabla_{\theta }\text{J(}\theta \text{)}={\text{E}}_{\pi \theta }\left[\sum_{\text{t}=\text{0}}^{\text{T}}\nabla_{\theta }{log\pi }_{\theta }\left({\text{a}}_{\text{t}}|{\text{s}}_{\text{t}}\right)\cdot {\text{Q}}^{\pi }\left({\text{s}}_{\text{t}},{\text{a}}_{\text{t}}\right)\right] \end{array}$$ (29)

To mitigate the high variance issue inherent in Monte Carlo policy gradient estimates, we employ the advantage function ${\text{A}}^{\pi }\left({\text{s}}_{\text{t}},{\text{a}}_{\text{t}}\right)={\text{Q}}^{\pi }\left({\text{s}}_{\text{t}},{\text{a}}_{\text{t}}\right)-{\text{V}}^{\pi }\left({\text{s}}_{\text{t}}\right)$ as a bias-free variance reduction technique [30]. Specifically, ${\text{V}}^{\pi }{\text{(s}}_{\text{t}}\text{)}$ serves as a state-dependent baseline (also known as the critic) that estimates the expected return starting from state s, while ${\text{A}}^{\pi }{\text{(s}}_{\text{t}},{\text{a}}_{\text{t}}\text{)}$ quantifies the relative advantage of action a compared to the average performance at state s. This baseline subtraction significantly reduces the variance of gradient estimates without introducing bias, thereby preventing policy collapse in high-dimensional parameter optimization [31]. In practice, ${\text{V}}^{\pi }{\text{(s}}_{\text{t}}\text{)}$ is approximated using the mean fitness of the current population as an unbiased estimator, which has been verified effective for maintaining convergence stability in our 6-dimensional continuous control scenario.

Based on Monte Carlo sampling, the gradient can be approximated as:


$$\begin{array}{c} \nabla_{\theta }\text{J(}\theta \text{)}\approx \frac{\text{1}}{\text{N}}\sum_{\text{i}=\text{1}}^{\text{N}}\sum_{\text{t}=\text{0}}^{T}\nabla_{\theta }\text{log}\pi_{\theta }\left(\overset{\text{(i)}}{\underset{\text{t}}{\text{a}}}|\overset{\text{(i)}}{\underset{\text{t}}{\text{s}}}\right)\cdot \overset{\text{(i)}}{\underset{\text{t}}{\text{R}}} \end{array}$$ (30)

Among them, N is the number of sampled trajectories and $\overset{\text{(i)}}{\underset{\text{t}}{\text{R}}}=\sum_{\text{k}=\text{t}}^{\text{T}}\gamma^{\text{k}-\text{t}}\overset{\text{(i)}}{\underset{\text{k}}{\text{r}}}$ is the discounted return. In practical implementation, Adam optimizer is used for stable updates.

The gradient formula has been clarified, and a complete training algorithm pseudocode will be provided for engineering implementation. Algorithm 2 provides the complete training process for PG-Tuner, ensuring that the policy learning process strictly corresponds to the DE evolution dynamics.

**Algorithm 2** **PG-Tuner training algorithm based on policy gradient**

Input:

 • Policy network θ (fully connected neural network, randomly initialized)

 • Training function set M

 • The number of trajectories per function L

 • Number of iterations per trajectory T

 • Population size NP

 • Learning rate α

 • Discount factor γ

Output:

 • Trained policy network parameters θ

1: for epoch = 1 to MaxEpoch do

2:  for each function fm in M do

3:   Randomly initialize population P_0_ and calculate fitness f_0_

4:   for l = 1 to L do

5:    t ← 0

6:    repeat

7:     Build state s_t_ (according to formula 24)

8:     Forward propagation: $\mu_{\text{t}},log\sigma \leftarrow \pi_{\theta }\left({\text{s}}_{\text{t}}\right)$(calculated from left to right according to the data in Fig 3)

9:     Sampling action${\text{a}}_{\text{t}}\leftarrow \text{N}\left(\mu_{\text{t}},\sigma^{\text{2}}\right)$

10:    Crop action to legal range (${\text{a}}_{\text{t}}\in \text{(0,1)}$)

11:    Execute DE Generation 1:${\text{P}}_{\text{t}+\text{1}}\leftarrow \text{DE}\left({\text{P}}_{\text{t}};{\text{a}}_{\text{t}}\right)$

12:    Calculate the reward r_t_ (according to formula 27)

13:    Storage experience${\text{(s}}_{\text{t}},{\text{a}}_{\text{t}},{\text{r}}_{\text{t}}\text{)}$

14:    t ← t + 1

15:   until t ≥ T

16:  end for

17:  Update θ using REINFORCE (according to formula 30)

18:  end for

19:end for

20:return θ

Explanation: State construction, action sampling, and reward calculation all correspond strictly to the previous MDP modeling, ensuring that policy learning is evidence-based.

After training, embed the obtained policy network θ into the main loop, so that the six dimensional parameters are generated in real-time for each generation and updated synchronously with DE evolution. The trained PG-Tuner has been integrated into the main loop of Algorithm 3. The embedding logic corresponds to the following:

(1) Each generation’s six dimensional parameters are generated in real-time by π_θ_ – lines 14–16;
(2) Six dimensional parameter driven algorithm two-stage mutation/crossover operation – lines 20–23;
(3) Environmental feedback rewards and online fine-tuning of π_θ_ every 10 generations – lines 28–32.

### 3.4. Algorithm flow

Algorithm 3 provides the complete process of RL-DE, clearly demonstrating the integration of PG-Tuner, two-stage mutation, and LB-Refiner.

**Algorithm 3** **RL-DE main algorithm process**

Input:

 • Objective function f(x)

 • Population size NP

 • Maximum iteration times T

 • L-BFGS activation probability LS_P

 • Reinforcement learning policy network parameter θ

Output:

 • Optimal solution x_best_

1: # Initialize

2: Randomly initialize population P_0_={x_1_, x_2_,..., x_NP_}

3: Initialize policy network π_θ_ (randomly initialized)

4: Initialize external archive set A=∅

5: Initialize reward accumulator R_accum_ = 0

6: for t = 1 to T do

7: # PG-Tuner observes the current population status:

8: Construct state vector s_t_ (according to formula 24)

9:  Forward propagation: $\mu_{\text{t}},log\sigma \leftarrow \pi_{\theta }{\text{(s}}_{\text{t}}\text{)}$ (calculated from left to right according to the data in Fig 3)

10: Sampling action${\text{a}}_{\text{t}}\leftarrow \text{N}\left(\mu_{\text{t}},\sigma^{\text{2}}\right)$

11: # DE engine executes a generation of evolution

12: if t ≤ 0.5 * MaxCycle then

13:  Using the DE/rand/2 + A strategy (Formula 16) for mutation

14: else

15:  Use DE/current to pBest-w/1 + A strategy (Formula 17) for mutation

16: Perform cross operation to generate test vectors

17: Perform selection operation to update population P_t_

18: Update external archive set A based on probability (as described in section 3.1)

19: # Calculate rewards and update strategies

20: Calculate the immediate reward r_t_ (according to formula 27)

21: Calculate cumulative rewards Raccum ← Raccum+r_t_

22: If t mod 10=0 then use policy gradient to update π_θ_ (according to algorithm 1)

23: # Check LB-Refiner activation

24: if t ≥ 0.9 * T and rand() <LS_P then

25:  Select the current optimal solution x_best_ and a random individual from the top 5% elite individual as the initial point for L-BFGS

26: Perform L-BFGS local optimization to obtain x_refined_

27: if f(x_refined_)<f(x_best_) then

28:   x_best_ ← x_refined_

29:end for

30:Return the optimal solution x_best_

Instructions:

(1) State action reward closed loop: Each generation’s state construction, action sampling, and reward calculation strictly correspond to the previous MDP modeling;
(2) Policy network embedding: The decision output (a_t_) of PG-Tuner directly drives the parameters in mutation formulas 16 and 17;
(3) Online fine-tuning: policy updates are performed every 10 generations to adapt to the current problem characteristics;
(4) Resource allocation: L-BFGS is only activated in the last 10% of iterations to avoid premature consumption of computational budget.

## 4. Experimental setup and results

### 4.1. Benchmark function suite

In order to rigorously evaluate the generalization performance of the proposed RL-DE framework, the experiment used a completely separate training-test benchmark partition: the policy network training phase only used the Congress on Evolutionary Computation 2014 (CEC2014) suite (30 functions) as the training set; The performance evaluation phase is conducted on the CEC 2017 suite (29 functions) without overlapping instances, fundamentally eliminating the risk of information leakage. The CEC 2017 suite includes unimodal, multimodal, mixed, and composite feature functions with domains of [−100, 100] and dimensions set to 10, 30, 50, and 100 to test the algorithm’s scalability. To ensure reproducibility and fair comparison, all experiments in Section 4 employ consistent settings across all dimensions: population size NP = 100, maximum function evaluations MaxFES = 5000 × D (where D is the problem dimension), and 30 independent runs for each algorithm on each test function.

To further validate the robustness of RL-DE on more recent and challenging multimodal landscapes, and to address potential concerns regarding overfitting to the CEC2017 characteristics, we additionally evaluate the proposed framework on the IEEE CEC2022 benchmark suite. The CEC2022 suite comprises 12 composite functions with complex terrain features specifically designed to test algorithm scalability in 10-dimensional and 20-dimensional spaces. Following the standard experimental protocol, we set MaxFES = 200,000 for D = 10 and MaxFES = 1,000,000 for D = 20, conducting 30 independent runs per function. Comparative analyses against SaDE, SHADE, ILSHADE, JSO, MPEDE, and LSHADE demonstrate that RL-DE maintains consistent performance advantages on this newer benchmark, corroborating the high-dimensional robustness claims presented in Section 4.2. Detailed numerical results, including mean values, standard deviations, and convergence curves, are provided in S5 and S6 Tables of the Supporting Information.

### 4.2. Comparison and analysis of effects

To comprehensively evaluate the performance of the proposed RL-DE framework, this section compares and analyzes the comprehensive performance of RL-DE with six representative differential evolution variants (DE, SaDE, SHADE, LSHADE, ILSHADE, jSO, MPEDE) on the CEC2017 test suite from three dimensions: solution accuracy, convergence speed, and statistical significance. The experiment covers four dimensions: 10, 30, 50, and 100, and each configuration is independently run 30 times to ensure the statistical reliability of the results.

#### 4.2.1. Accuracy comparison and analysis.

Tables 1-4 show the average optimal values obtained by each algorithm on 29 test functions of CEC2017 in 10, 30, 50, and 100 dimensions (average of 30 independent runs). To facilitate comparison, all optimal values are kept in the Scientific notation format, and the algorithm results showing the best performance on each function are marked in bold (the complete data including standard deviations are provided in S1-S4 Tables).

As shown in Table 1, for the 10-dimensional CEC2017 functions, all algorithms achieve identical optimal values on F2 and F5, which are therefore excluded from the comparative analysis. Among the remaining 27 functions, RL-DE ranks first on 11 functions, followed by LSHADE with 7 functions, while MPEDE, ILSHADE, and JSO each secure the top position on 5 functions. Notably, RL-DE and LSHADE demonstrate the strongest overall performance, ranking as the top two algorithms in the 10-dimensional test suite.

**Table 1:** Comparison of optimal values for 10 dimensional CEC2017 functions (30 runs).

Function	DE	SaDE	SHADE	ILSHADE	JSO	MPEDE	LSHADE	RL-DE	best_value
**F1**	133.295	100.005	**100**	**100**	**100**	174.752	**100**	**100**	100
**F2**	200	200	200	200	200	200	200	200	200
**F3**	304.068	303.321	303.059	303.455	304.18	302.931	**302.392**	302.923	300
**F4**	418.598	408.116	407.548	402.91	403.104	415.546	**402.214**	404.776	400
**F5**	500	500	500	500	500	500	500	500	500
**F6**	630.681	619.687	618.485	613.064	613.538	627.085	**612.122**	615.22	600
**F7**	758.4	761.2	764	771	772	730.82	753.2	**716.487**	700
**F8**	802.153	802.122	802.176	802.254	802.326	801.848	801.548	**800**	800
**F9**	1882.445	2135.399	2358.001	2616.066	2708.886	1168.215	1840.955	**1128.822**	900
**F10**	1040.871	1055.83	1026.748	**1000.36**	1001.283	1070.712	1000.945	1002.592	1000
**F11**	4215.704	1193.944	1129.713	1163.91	**1114.615**	1224.352	1149.537	1134.967	1100
**F12**	1469.065	1209.989	1208.483	1207.317	1204.983	1275.708	2246.568	**1204.936**	1200
**F13**	1340.032	1330.262	1326.753	1319.969	1319.343	1332.342	1317.932	**1316.206**	1300
**F14**	1415.022	1402.819	1402.011	**1400.302**	1400.759	1411.333	1465.235	1400.611	1400
**F15**	1500.579	1518.133	1521.127	1564.982	1631.879	1502.042	**1500.394**	1529.235	1500
**F16**	1637.865	1640.451	1653.398	1701.432	1673.777	1641.697	**1621.683**	1640.605	1600
**F17**	1725.061	1713.116	1712.313	1714.377	1712.347	1719.457	1706.459	**1703.964**	1700
**F18**	1803.495	1801.92	1802.201	1801.375	1801.26	1804.066	**1800.767**	1801.831	1800
**F19**	1910.406	**1903.027**	1903.578	1916.755	1910.093	1920.985	1903.261	1923.075	1900
**F20**	2166.419	2162.892	2164.476	2134.105	2144.934	**2100**	2140.944	**2100**	2000
**F21**	2446.348	2395.947	2556.558	2837.849	2895.205	**2200.01**	2200.699	2200.046	2100
**F22**	2310	2343.333	2333.333	2306.667	2316.667	**2303.339**	2466.719	2306.667	2200
**F23**	**2400**	**2400**	**2400**	**2400**	**2400**	2400.001	2410	2500	2300
**F24**	2905.171	2901.921	2913.277	2913.598	2911.617	2902.267	2883.701	**2878.872**	2400
**F25**	**2500**	2631.161	2780.048	2570.126	2535.426	2531.151	2830.393	2963.09	2500
**F26**	3123.193	3027.101	3064.062	3199.365	3248.278	**2990.332**	2997.836	2992.563	2600
**F27**	**2700**	**2700**	**2700**	**2700**	**2700**	**2700**	2850.753	**2700**	2700
**F28**	3879.061	4177.111	4397.992	3930.268	4875.53	3833.623	4870.203	**3831.846**	2800
**F29**	9967.824	6523.956	4862.893	4876.545	**4261.931**	4988.963	4669.055	4774.298	2900

As shown in Table 2, among the 29 functions, all algorithms achieve identical optimal values on F2 and F5, which is excluded from the count. Among the remaining 27 functions, the RL-DE algorithm ranks first in 12 functions; the MPEDE algorithm ranks first in 8 functions; and the JSO algorithm ranks first in 6 functions. Notably, RL-DE, MPEDE, and JSO demonstrate the strongest overall performance, securing the top three positions in the 30-dimensional CEC2017 function test.

**Table 2:** Comparison of optimal values for 30 dimensional CEC2017 functions (30 runs).

Function	DE	SaDE	SHADE	ILSHADE	JSO	MPEDE	LSHADE	RL-DE	best_value
**F1**	7308.822	100.349	**100**	100.002	230.604	**100**	**100**	**100**	100
**F2**	200	200	200	200	200	200	200	200	200
**F3**	333.903	**315.155**	329.894	333.222	336.136	329.317	328.108	319.086	300
**F4**	582.45	461.396	438.323	445.058	**431.131**	478.115	446.057	432.834	400
**F5**	500.001	500.001	**500**	**500**	**500**	**500**	500	**500**	500
**F6**	818.456	695.47	672.261	680.202	**663.128**	719.925	689.737	664.944	600
**F7**	1153.938	1159.401	1189.167	1175.483	1171.817	1135.399	1168.45	**831.505**	700
**F8**	809.192	810.793	811.151	811.382	811.221	805.521	810.366	**800.223**	800
**F9**	4344.396	4862.49	5230.96	5144.541	5168.252	4635.275	5005.23	**3515.911**	900
**F10**	49188.376	2421.893	1350.948	1506.536	1215.437	2838.429	1753.788	**1174.531**	1000
**F11**	7119885.193	5417.947	5517.148	5914.533	4230.408	8227.979	4334.131	**2457.553**	1100
**F12**	18535.513	3324.325	2394.624	3287.163	1798.422	1658.004	3181.854	**1388.096**	1200
**F13**	23095.17	2164.958	1698.024	1805.406	1721.44	1667.971	1987.943	**1463.704**	1300
**F14**	85651.784	7712.177	3078.857	3790.198	3057.646	3275.967	3015.086	**2292.068**	1400
**F15**	1580.399	1555.281	1558.668	1718.712	1634.247	**1547.182**	1682.727	1621.824	1500
**F16**	5748.633	1803.455	1856.056	1815.354	1736.227	**1711.079**	1781.768	1758.303	1600
**F17**	34001.465	4232.033	2865.796	2192.714	2136.144	3128.797	33357.484	**1751.297**	1700
**F18**	3393.493	1877.986	1818.838	1815.771	**1814.399**	2562.812	1816.209	1815.165	1800
**F19**	2210.428	2154.085	2135.17	2101.501	2135.802	**2041.622**	2081.035	2067.321	1900
**F20**	2138.345	2234.18	2231.699	2138.3	2149.874	2149.46	2182.361	**2128.806**	2000
**F21**	2798.606	2653.261	3894.048	4897.218	5261.024	**2262.725**	2308.458	2271.162	2100
**F22**	**2400**	2411.842	2405.003	2412.604	2401.831	**2400**	2445.66	2404.545	2200
**F23**	**2500**	2515.801	2508.726	2527.727	2522.223	2505.602	2500	**2500**	2300
**F24**	2821.555	2822.016	2824.605	2826.088	2825.894	**2820.813**	2821.01	2821.618	2400
**F25**	3259.635	3276.745	3227.253	3238.589	**3191.551**	3327.943	3339.201	3320.403	2500
**F26**	**3102.166**	3535.629	4176.688	5341.306	5879.4	3104.443	3185.941	3114.591	2600
**F27**	2700.001	**2700**	**2700**	2707.181	**2700**	**2700**	3268.006	2701.406	2700
**F28**	48717.928	5657.771	5620.706	6377.201	**5428.218**	5571.067	6347.894	6792.285	2800
**F29**	148988.863	7687.043	**5468.738**	6818.403	6162.62	5811.002	5879.448	5787.54	2900

As shown in Table 3, for the 50-dimensional CEC2017 functions, all algorithms achieve identical optimal values on F2 and F5, which are therefore excluded from the count. Among the remaining 27 functions, the RL-DE algorithm ranks first in 13 functions; the SHADE algorithm ranks first in 4 functions; and the SaDE algorithm ranks first in 4 functions. The RL-DE, SHADE, and SaDE algorithms demonstrate the strongest overall performance, securing the top three positions in the 50-dimensional test suite.

**Table 3:** Comparison of optimal values for 50 dimensional CEC2017 functions (30 runs).

Function	DE	SaDE	SHADE	ILSHADE	JSO	MPEDE	LSHADE	RL-DE	best_value
**F1**	1105.695	2406.941	1516.455	2254.039	3473.015	100.278	2751.882	**100**	100
**F2**	209.551	200	200	200	200	200	200	200	200
**F3**	455.714	376.559	391.07	417.534	426.824	372.576	418.376	**327.472**	300
**F4**	802.408	528.979	**494.154**	552.759	513.832	569.825	574.467	526.757	400
**F5**	500.003	500.001	500.001	500.001	500.001	500.001	500	500	500
**F6**	1054.98	800.965	**766.171**	810.159	774.6	842.743	853.023	792.233	600
**F7**	1557.898	1541.085	1561.9	1562.733	1566.133	1099.739	1413.934	**910.517**	700
**F8**	818.709	822.008	822.552	822.55	821.94	814.887	817.018	**803.466**	800
**F9**	7858.849	9068.693	9336.862	9293.533	9280.918	8014.411	9434.775	**6910.069**	900
**F10**	253234.689	3749.413	6351.591	6522.327	2926.916	3589.144	3615.807	**1367.97**	1000
**F11**	48718125.4	13174.364	12800.639	12538.826	15742.203	10444.873	12942.154	**3059.68**	1100
**F12**	59723.275	13824.966	10843.029	18942.581	19205.434	15293.228	11667.3	**1411.537**	1200
**F13**	166398.616	10970.311	17609.726	9557.652	7549.286	8460.497	11375.07	**1808.91**	1300
**F14**	274172.739	6203.061	6623.683	7998.304	9971.52	5331.259	8184.187	**2916.645**	1400
**F15**	3068.331	1983.333	**1873.701**	2181.07	2106.489	1919.699	1940.252	2146.526	1500
**F16**	23583.355	**1788.428**	1857.441	2071.95	1913.105	1873.168	2082.243	2032.052	1600
**F17**	341094.414	13654.629	10034.852	4850.206	8844.265	16436.884	16173.644	**1852.773**	1700
**F18**	13186.31	3500.512	3910.249	2732.217	**1846.665**	2045.336	2124.672	3285.503	1800
**F19**	2078.541	2018.677	**2008.765**	2036.079	2018.8	2021.821	2027.816	2013.349	1900
**F20**	2437.945	2227.37	2238.949	2274.106	2230.481	2274.299	**2188.581**	2198.396	2000
**F21**	2434.986	3869.772	7315.856	8493.072	8726.193	**2298.171**	3828.167	2342.9	2100
**F22**	2864.531	2574.627	2540.825	2642.021	2600.55	2625.769	2689.245	**2406.767**	2200
**F23**	2777.933	2635.026	2620.489	2600.57	2612.652	2621.969	2610.561	**2506.217**	2300
**F24**	**3158.774**	3214.245	3223.878	3259.395	3202.573	3191.305	3182.27	3173.622	2400
**F25**	3752.789	**3608.237**	3652.913	3701.659	3688.425	3829.276	4248.646	3741.175	2500
**F26**	**3108.405**	6095.065	4955.888	8662.425	9104.332	3129.493	3529.61	3169.77	2600
**F27**	2700.002	**2700**	2737.956	2772.934	2741.371	**2700**	2969.354	2747.352	2700
**F28**	492527.518	**8349.474**	8628.144	9132.514	8725.594	8546.655	8913.904	8701.305	2800
**F29**	72342938.7	48327.044	25867.796	27827.113	**21581.8**	22908.329	25434.018	23818.191	2900

As shown in Table 4, among the 29 functions, all algorithms achieve identical optimal values on F5, which are therefore excluded from the count. Among the remaining 28 functions, the RL-DE algorithm ranks first in 14 functions; the SaDE algorithm ranks first in 6 functions; and the SHADE algorithm ranks first in 5 functions. The RL-DE, SaDE, and SHADE algorithms demonstrate the strongest overall performance, securing the top three positions in the 100-dimensional CEC2017 function test.

**Table 4:** Comparison of optimal values for 100 dimensional CEC2017 function (30 runs).

Function	DE	SaDE	SHADE	ILSHADE	JSO	MPEDE	LSHADE	RL-DE	best_value
**F1**	3900.295	4699.795	5055.729	5039.926	5306.169	174.615	6419.604	**100**	100
**F2**	9266.835	**200**	**200**	**200**	200.004	**200**	202.203	**200**	200
**F3**	593.592	517.876	518.291	529.672	565.923	506.746	543.308	**315.337**	300
**F4**	1449.152	828.775	**776.914**	1117.884	895.261	972.105	1395.041	1109.592	400
**F5**	500.012	500	500	500	500	500.001	500	500	500
**F6**	1760.818	1152.718	**1135.878**	1542.555	1221.225	1288.146	1520.436	1412.907	600
**F7**	2655.427	2435.55	2558.7	2553.567	2506.067	2380.063	2528.75	**1308.983**	700
**F8**	882.996	860.86	860.621	861.607	860.507	849.77	858.827	**819.592**	800
**F9**	27645.153	18105.11	17920.829	18338.012	18389.356	20690.933	19467.29	**15023.131**	900
**F10**	14626.097	1481.61	1380.44	2084.968	2063.365	1434.548	1956.421	**1289.026**	1000
**F11**	1272493911	21849.588	22639.888	121597.618	209529.149	18833.17	71289.21	**3475.769**	1100
**F12**	11605.204	14328.692	11856.168	16429.47	11550.368	10161.805	16429.96	**1727.14**	1200
**F13**	966605.639	38054.518	18982.436	39983.862	37005.816	35393.683	68509.717	**2175.245**	1300
**F14**	148646.015	9781.662	12763.307	15744.846	16765.031	12430.247	13184.271	**2890.077**	1400
**F15**	6738.339	1809.248	1995.576	4478.331	5751.736	**1922.739**	3755.134	2246.084	1500
**F16**	30434867.23	2432.555	2973.089	3618.983	3223.873	3474.273	3780.422	**2367.948**	1600
**F17**	5148773.258	40156.63	30956.29	17893.614	25720.378	40806.078	22335.731	**2435.134**	1700
**F18**	8801.056	**3187.674**	3321.262	9627.831	6027.952	3315.368	6247.76	6264.258	1800
**F19**	2408.201	**2004.849**	2035.231	2673.414	2388.742	2011.297	2096.394	2118.081	1900
**F20**	3262.013	2592.885	**2589.129**	2926.517	2701.687	2736.818	3039.953	2802.054	2000
**F21**	**2200.014**	5371.521	14287.285	15277.28	15287.985	2329.169	6750.148	2435.419	2100
**F22**	**2473.82**	2501.604	2497.694	2582.105	2680.509	2507.21	2667.4	2514.13	2200
**F23**	2575.171	2692.196	2843.823	2750.425	2703.19	2596.389	2675.639	**2517.608**	2300
**F24**	**3279.918**	3306.312	3346.237	3360.054	3371.13	3321.187	3368.861	3354.014	2400
**F25**	5834.637	3580.791	**3211.493**	4760.706	4346.723	5759.624	5784.613	5420.057	2500
**F26**	3356.993	4870.281	6183.636	10584.544	11976.004	**3283.435**	3627.47	3356.666	2600
**F27**	3207.03	**2705.909**	2730.038	2806.991	2752.869	2722.606	2814.175	2799.384	2700
**F28**	15443986.88	**11063.881**	12057.037	15097.623	13812.103	11105.036	15679.671	14406.032	2800
**F29**	2655853.526	**25764.743**	28581.227	50817.94	40075.895	26357.458	42138.22	38079.185	2900

To quantitatively summarize the performance advantages of RL-DE, we conducted a comprehensive statistical analysis of the ranking results across all dimensions. Tables 1-4 show that RL-DE achieved the best performance on 11/27 (40.7%), 12/27 (44.4%), 13/27 (48.1%), and 14/28 (50.0%) of functions in 10D, 30D, 50D, and 100D, respectively (with F2 and F5 excluded from the 10D-50D comparisons as all algorithms achieved identical optimal values on these functions, and F5 excluded from the 100D comparison). Furthermore, supported by the Wilcoxon signed-rank test results presented in Section 4.2.3, RL-DE demonstrates statistically significant superiority (p < 0.05) over baseline algorithms on the majority of functions. Additionally, the function-level statistical significance analysis in Table 10 reveals that RL-DE achieves statistically significant superiority over traditional DE on 70.4%−81.5% of functions across the four dimensions, with the proportions of significant superiority over other adaptive DE variants (SaDE, SHADE, LSHADE, ILSHADE, jSO, MPEDE) ranging from 44.4% to 70.4%. These quantitative metrics, combined with the Friedman test results showing RL-DE consistently ranking first across all dimensions, validate the robustness of its performance advantage from both ranking-based and statistical significance perspectives.

#### 4.2.2. Comparison and analysis of convergence speed.

To visually evaluate the convergence behavior of RL-DE and seven other comparison algorithms (DE, SaDE, SHADE, LSHADE, ILSHADE, jSO, MPEDE) during the search process, this section selects a representative function F23 from the CEC2017 test function set and plots its convergence curve in 10, 30, 50, and 100 dimensions. F23 is a multimodal composite function with complex terrain and multiple local extrema, which can effectively test the algorithm’s ability to explore and balance development in different dimensions.

**Fig 4:**
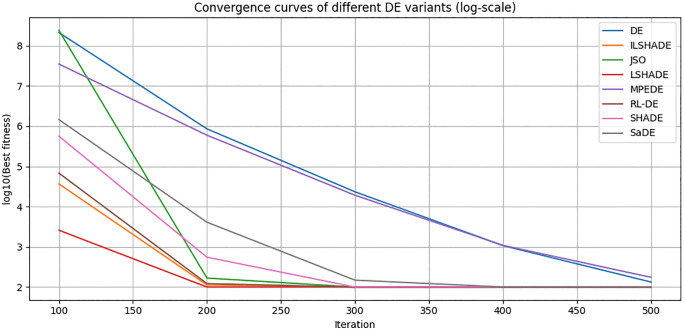
10 dimensional F23 function convergence curve (average).

**Fig 5:**
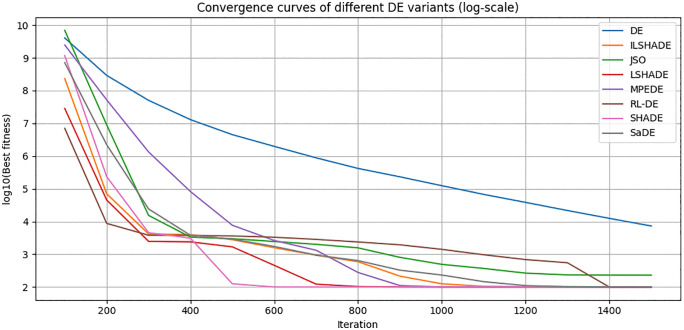
30 dimensional F23 function convergence curve (average).

**Fig 6:**
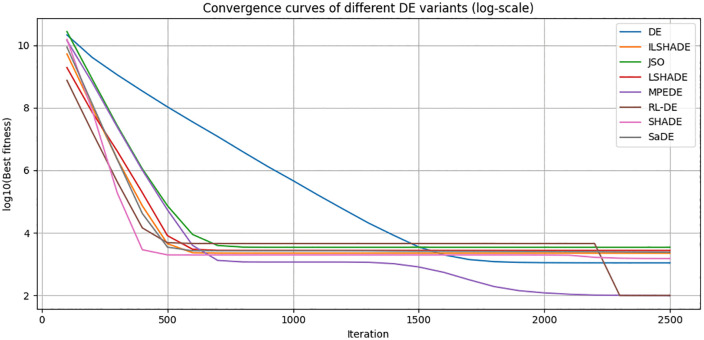
50 dimensional F23 function convergence curve (average).

**Fig 7:**
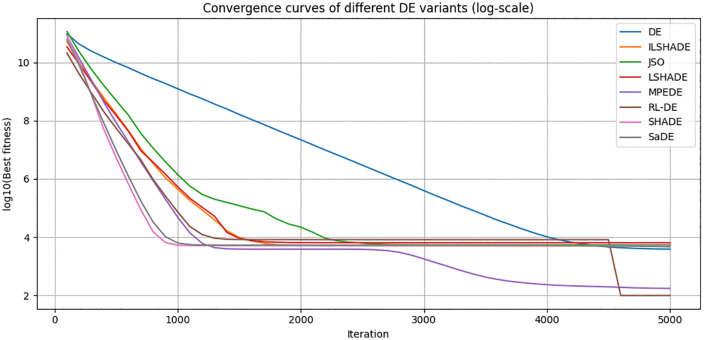
100 dimensional F23 function convergence curve (average).

The horizontal axis in the figure represents the number of function evaluations (recorded every 100 times), and the vertical axis represents the current optimal solution error value (taking the average of 30 independent runs, using logarithmic coordinates to highlight differences). Figs 4–7 respectively show the convergence trends of the F23 function in four dimensions.

From the figure, it can be observed that:

(1) The convergence speed in the early stage varies significantly with dimensionality
In the 10 dimensional stage, LSHADE, ISHADE, and RL-DE rank among the top three; At 30 dimensions, RL-DE ranks first, followed closely by LSHADE and ISHADE, indicating that the policy gradient controller can enter the efficient search phase ahead of schedule in the medium dimension; In 50 dimensions, SHADE achieved first place, while RL-DE and SaDE ranked second and third respectively; After entering the 100 dimensions, SHADE, SaDE, and MPEDE occupy the top three, while RL-DE ranks fourth.
(2) Outstanding ability to continuously decline in the later stage
Although RL-DE starts up slightly slower than some comparison algorithms in the high-dimensional early stage, its error curve can still maintain a stable decrease after 90% evaluation budget, verifying the improvement effect of LB-Refiner on the final solution accuracy.
(3) High dimensional adaptability gradually emerges
As the dimensionality increases, although the RL-DE ranking fluctuates in the early stage, its later convergence stability is better than most comparison algorithms, reflecting the robustness of the reinforcement learning parameter controller in complex terrain.

In summary, RL-DE exhibits a convergence feature of competitive in the early stage and stable decline in the later stage on F23. Its six parameter adaptive mechanism based on policy gradient and L-BFGS local reinforcement module work together to effectively balance global exploration and local development, and maintain continuous evolution ability in complex multimodal and high-dimensional scenarios.

#### 4.2.3. Robustness comparison and analysis.

To evaluate the stability and consistency of RL-DE in multiple runs, this paper conducted non parametric Friedman test and paired Wilcoxon rank test (α = 0.05) on the average optimal values of 29 functions and 30 independent runs of each function in CEC2017. The experiment covers 10, 30, 50, and 100 dimensions, and the results are as follows:

(1) Overall ranking: Friedman test

The results in Table 5 show that the Friedman test gives p < 0.01 for all four dimensions (specifically, p = 3.85 × 10^-3^ for 10D, p = 6.28 × 10^-7^ for 30D, p = 3.46 × 10^-9^ for 50D, and p = 6.56 × 10^-14^ for 100D), indicating statistically significant differences between the algorithms. Further observation of the average rank reveals that:

**Table 5:** Friedman average ranking and overall ranking (10/30/50/100 dimensions).

Dimension	p-value	1st	Mean Rank	2nd	Mean Rank	3rd	Mean Rank
**10**	3.85e-03	RL-DE	3.17	LSHADE	3.62	MPEDE	4.60
**30**	6.28e-07	RL-DE	2.57	MPEDE	3.50	JSO	4.26
**50**	3.46e-09	RL-DE	2.40	MPEDE	3.57	SHADE	4.21
**100**	6.56e-14	RL-DE	2.88	SaDE	2.98	MPEDE	3.21

RL-DE always maintains first place:

Starting from the 10-dimensional scenario, it ranks first with an average rank of 3.17, and then further improves to 2.57 and 2.40 in the 30-dimensional and 50-dimensional tests, respectively, indicating that as the dimension increases, its relative advantage actually expands; Although it has slightly rebounded to 2.88 at 100 dimensions, it still firmly holds the top spot.

Other algorithms drift with dimensionality, while RL-DE maintains a stable position:

In 10 dimensions, LSHADE (3.62) and MPEDE (4.60) are ranked second and third, respectively; After 30 dimensions, MPEDE remained stable in the top three with average ranks of 3.50 (30D), 3.57 (50D), and 3.21 (100D), demonstrating good cross-dimensional stability; SaDE jumped to second place (2.98) in 100 dimensions, trailing RL-DE by 0.10, indicating that its adaptive mechanism has also begun to exert force in high-dimensional scenarios, but it has not yet surpassed RL-DE.

In summary, the Friedman test consistently confirms from an overall ranking perspective that the six parameter adaptive mechanism of policy gradient proposed by RL-DE can provide the most stable and significantly optimal solution quality in the 10–100 dimensional range, laying a solid robustness foundation for its subsequent application in real high-dimensional engineering problems.

(2) Pairwise significance: Wilcoxon signed-rank test

To verify whether RL-DE is significantly superior to other comparison algorithms, this study employed the Wilcoxon signed-rank test for pairwise comparisons. This non-parametric test is specifically designed for paired samples, where each algorithm’s performance on the same test function constitutes a natural pair, effectively controlling for inter-function variability and isolating algorithmic performance differences. The test evaluates whether the median performance difference across the 29 benchmark functions (each assessed via 30 independent runs) significantly deviates from zero, with the null hypothesis stating no difference between algorithms and the alternative hypothesis stating RL-DE performs significantly better. Statistical significance is determined at the α = 0.05 level.

As shown in Table 6, in the 10 dimensional scenario, RL-DE achieved p < 0.05 compared to traditional DE, ILSHADE, MPEDE, SHADE, and SaDE, indicating significant differences; Compared with jSO, p = 0.135 > 0.05, The difference between the two has not yet reached a significant level, but the average rank of RL-DE is still lower than that of jSO, indicating that its overall solution quality is better. Entering the 30th dimension (Table 7), the significant advantages of RL-DE are further expanded: except for MPEDE which is close to it in some functions (p = 0.023), RL-DE is significantly better than other algorithms, especially with a p-value lower than DE of 2.16 × 10^−4^, indicating that as the dimension increases, the performance gain brought by RL-DE’s policy gradient six parameter adaptive mechanism becomes more significant.

**Table 6:** Pairwise Wilcoxon signed-rank p-value matrix (paired-sample test, < 0.05 indicates significant difference) (10 dimensions).

	DE	ILSHADE	JSO	LSHADE	MPEDE	SHADE	SaDE	RL-DE
**DE**	1.00e + 00	8.40e-01	8.61e-01	2.20e-01	2.01e-02	6.00e-01	2.42e-01	**5.01e-03**
**ILSHADE**		1.00e + 00	5.49e-01	4.39e-01	6.75e-01	6.07e-01	7.16e-01	**2.07e-02**
**JSO**			1.00e + 00	5.85e-01	6.94e-01	9.54e-01	9.25e-01	1.35e-01
**LSHADE**				1.00e + 00	6.48e-01	4.54e-01	3.02e-01	2.28e-01
**MPEDE**					1.00e + 00	5.01e-01	4.09e-01	**4.80e-02**
**SHADE**						1.00e + 00	4.76e-01	**1.60e-02**
**SaDE**							1.00e + 00	**7.95e-03**
**RL-DE**								1.00e + 00

**Table 7:** Pairwise Wilcoxon signed-rank p-value matrix (paired-sample test, < 0.05 indicates significant difference) (30 dimensions).

	DE	ILSHADE	JSO	LSHADE	MPEDE	SHADE	SaDE	RL-DE
**DE**	1.00e + 00	2.56e-02	2.15e-02	8.83e-03	5.41e-04	1.01e-02	3.94e-03	**2.16e-04**
**ILSHADE**		1.00e + 00	3.25e-02	2.69e-01	1.36e-01	2.25e-02	7.16e-01	**1.78e-04**
**JSO**			1.00e + 00	3.37e-01	7.32e-01	3.54e-01	1.02e-01	**2.67e-03**
**LSHADE**				1.00e + 00	7.34e-02	6.57e-01	1.94e-01	**1.01e-04**
**MPEDE**					1.00e + 00	7.57e-01	4.88e-02	**2.30e-02**
**SHADE**						1.00e + 00	1.86e-01	**4.63e-03**
**SaDE**							1.00e + 00	**7.51e-04**
**RL-DE**								1.00e + 00

The 50 dimensional results (Table 8) show that RL-DE outperforms the control algorithm in all aspects: p-values with DE, ILSHADE, JSO, LSHADE, SHADE, and SaDE are all less than 0.01, and p = 0.047 with MPEDE is also significantly critical. It is worth noting that in 100 dimensions (Table 9), although SaDE and SHADE closely follow RL-DE in Friedman mean rank, the Wilcoxon test still gave p = 0.22 and p = 0.11, which did not reach a significant level; On the other hand, DE, ILSHADE, LSHADE, and JSO continued to be significantly surpassed by RL-DE (p < 0.02). This phenomenon indicates that as the dimensionality continues to increase, the gap between RL-DE and the strongest baseline narrows, but still maintains statistical significance, reflecting the robust generalization ability of its reinforcement learning controller in high-dimensional space.

**Table 8:** Pairwise Wilcoxon signed-rank p-value matrix (paired-sample test, < 0.05 indicates significant difference) (50 dimensions).

	DE	ILSHADE	JSO	LSHADE	MPEDE	SHADE	SaDE	RL-DE
**DE**	1.00e + 00	5.57e-03	4.11e-03	9.88e-03	7.58e-06	3.51e-03	1.98e-03	**7.71e-07**
**ILSHADE**		1.00e + 00	4.00e-01	6.82e-01	1.52e-03	3.45e-02	1.36e-01	**4.58e-05**
**JSO**			1.00e + 00	7.50e-01	3.13e-01	2.90e-01	1.86e-01	**6.28e-03**
**LSHADE**				1.00e + 00	2.28e-03	5.24e-01	5.85e-01	**1.21e-04**
**MPEDE**					1.00e + 00	1.07e-01	1.07e-01	**4.76e-02**
**SHADE**						1.00e + 00	8.85e-01	**1.81e-03**
**SaDE**							1.00e + 00	**1.32e-03**
**RL-DE**								1.00e + 00

**Table 9:** Pairwise Wilcoxon signed-rank p-value matrix (paired-sample test, < 0.05 indicates significant difference) (100 dimensions).

	DE	ILSHADE	JSO	LSHADE	MPEDE	SHADE	SaDE	RL-DE
**DE**	1.00e + 00	2.03e-02	1.67e-03	3.51e-03	1.38e-06	1.17e-03	1.17e-03	**6.30e-07**
**ILSHADE**		1.00e + 00	3.62e-01	7.50e-01	9.60e-04	3.14e-04	8.14e-05	**1.90e-05**
**JSO**			1.00e + 00	6.65e-01	9.88e-03	8.16e-04	1.96e-03	**1.68e-03**
**LSHADE**				1.00e + 00	7.73e-05	5.86e-03	2.94e-04	**9.98e-06**
**MPEDE**					1.00e + 00	3.87e-01	7.50e-01	2.55e-01
**SHADE**						1.00e + 00	2.20e-01	1.07e-01
**SaDE**							1.00e + 00	2.20e-01
**RL-DE**								1.00e + 00

To further quantify the statistical advantages of RL-DE relative to baseline algorithms, we conducted function-level significance tests using Welch’s t-test on the mean and standard deviation of 30 independent runs for each function (α = 0.05). Unlike the Wilcoxon signed-rank test, the t-test performs statistical inference based on mean and standard deviation, which is suitable for evaluating significant differences in means between algorithms. Table 10 summarizes the function-level statistical significance comparison results between RL-DE and comparison algorithms across 10D-100D dimensions, including the number and percentage of functions categorized as ‘statistically significantly better’, ‘no significant difference’, and ‘statistically significantly worse’.

**Table 10:** Summary of Function-Level Statistical Significance Comparison Between RL-DE and Baseline Algorithms.

Dimension	Comparison	Significantly Better	No Significant Difference	Significantly Worse	Total Functions
10D	RL-DE vs DE	19/27 (70.4%)	6/27 (22.2%)	2/27 (7.4%)	27
10D	RL-DE vs SaDE	19/27 (70.4%)	6/27 (22.2%)	2/27 (7.4%)	27
10D	RL-DE vs SHADE	16/27 (59.3%)	9/27 (33.3%)	2/27 (7.4%)	27
10D	RL-DE vs ILSHADE	10/27 (37.0%)	11/27 (40.7%)	6/27 (22.2%)	27
10D	RL-DE vs JSO	12/27 (44.4%)	9/27 (33.3%)	6/27 (22.2%)	27
10D	RL-DE vs MPEDE	12/27 (44.4%)	11/27 (40.7%)	4/27 (14.8%)	27
10D	RL-DE vs LSHADE	12/27 (44.4%)	8/27 (29.6%)	7/27 (25.9%)	27
30D	RL-DE vs DE	19/27 (70.4%)	5/27 (18.5%)	3/27 (11.1%)	27
30D	RL-DE vs SaDE	19/27 (70.4%)	5/27 (18.5%)	3/27 (11.1%)	27
30D	RL-DE vs SHADE	17/27 (63.0%)	7/27 (25.9%)	3/27 (11.1%)	27
30D	RL-DE vs ILSHADE	18/27 (66.7%)	8/27 (29.6%)	1/27 (3.7%)	27
30D	RL-DE vs JSO	12/27 (44.4%)	12/27 (44.4%)	3/27 (11.1%)	27
30D	RL-DE vs MPEDE	12/27 (44.4%)	10/27 (37.0%)	5/27 (18.5%)	27
30D	RL-DE vs LSHADE	17/27 (63.0%)	10/27 (37.0%)	0/27 (0.0%)	27
50D	RL-DE vs DE	22/27 (81.5%)	2/27 (7.4%)	3/27 (11.1%)	27
50D	RL-DE vs SaDE	17/27 (63.0%)	6/27 (22.2%)	4/27 (14.8%)	27
50D	RL-DE vs SHADE	16/27 (59.3%)	6/27 (22.2%)	5/27 (18.5%)	27
50D	RL-DE vs ILSHADE	18/27 (66.7%)	9/27 (33.3%)	0/27 (0.0%)	27
50D	RL-DE vs JSO	16/27 (59.3%)	8/27 (29.6%)	3/27 (11.1%)	27
50D	RL-DE vs MPEDE	18/27 (66.7%)	3/27 (11.1%)	6/27 (22.2%)	27
50D	RL-DE vs LSHADE	19/27 (70.4%)	6/27 (22.2%)	2/27 (7.4%)	27
100D	RL-DE vs DE	22/28 (78.6%)	4/28 (14.3%)	2/28 (7.1%)	28
100D	RL-DE vs SaDE	13/28 (46.4%)	4/28 (14.3%)	11/28 (39.3%)	28
100D	RL-DE vs SHADE	14/28 (50.0%)	5/28 (17.9%)	9/28 (32.1%)	28
100D	RL-DE vs ILSHADE	19/28 (67.9%)	8/28 (28.6%)	1/28 (3.6%)	28
100D	RL-DE vs JSO	18/28 (64.3%)	5/28 (17.9%)	5/28 (17.9%)	28
100D	RL-DE vs MPEDE	13/28 (46.4%)	5/28 (17.9%)	10/28 (35.7%)	28
100D	RL-DE vs LSHADE	19/28 (67.9%)	9/28 (32.1%)	0/28 (0.0%)	28

Based on the function-level statistical significance analysis results in Table 10, the following conclusions can be drawn: (1) RL-DE’s advantage over DE is the most stable and significant: Across the 10D-100D range, RL-DE achieves statistically superior performance on 70.4%−81.5% of functions and never performs significantly worse than DE, validating the substantial improvement of the PG-Tuner parameter adaptation mechanism over traditional DE; (2) RL-DE’s advantage over LSHADE increases noticeably with dimensionality: rising from 44.4% (10D) to 70.4% (50D) and maintaining 67.9% (100D), demonstrating the adaptive advantage of the reinforcement learning controller in high-dimensional scenarios; (3) RL-DE never performs statistically significantly worse than ILSHADE and LSHADE in 50D and 100D tests (significantly worse ratio: 0%), showing its robust handling capability for complex multimodal problems; (4) Challenges and opportunities coexist in 100D: Although the advantage ratios over SaDE, SHADE, and MPEDE decrease (46.4%−50.0%), RL-DE maintains significant superiority ratios of 64.3%−78.6% over DE, ILSHADE, LSHADE, and JSO, indicating its competitiveness in ultra-high-dimensional problems. These function-level statistical significance data corroborate the overall results from Friedman and Wilcoxon tests, collectively validating the effectiveness and robustness of RL-DE’s parameter adaptation mechanism.

#### 4.2.4. Computational cost analysis and performance-efficiency trade-off.

Although RL-DE demonstrates superior optimization performance, the integration of PG-Tuner inevitably introduces additional computational overhead compared to traditional adaptive DE variants. To quantify this trade-off, we conducted a runtime comparison experiment between RL-DE (including both training and inference phases), SHADE, and MPEDE across 10, 30, 50, and 100 dimensions on the CEC2017 benchmark. All experiments were executed on a standard computing platform equipped with an Intel Core i5-1135G7 processor (2.4 GHz) and 16 GB RAM, implemented in Python.

As shown in Table 11, the RL-DE framework involves two distinct stages: policy network training (RL-DE-Train) and deployment inference (RL-DE-Inference). The training phase, conducted once per dimension on the CEC2014 training set, requires substantial computational investment (ranging from 456 seconds for 10D to 12,721 seconds for 100D) due to the extensive policy gradient updates and episode sampling. However, this constitutes a one-time offline cost; once trained, the policy network is fixed and deployed for inference without requiring retraining for new problem instances within the same dimensionality.

**Table 11:** Runtime comparison (seconds) for a single run across different dimensions.

Dimension	RL-DE-Train	RL-DE-Inference	SHADE	vs SHADE (%)	MPEDE	vs MPEDE (%)
10	456	515	429	20.05%	462	11.47%
30	1844	2979	1862	59.99%	1879	58.54%
50	3667	6282	4512	39.23%	4395	42.94%
100	12721	22717	13104	73.36%	10589	114.53%

The values denote the time required for one single execution. The statistical results in previous sections are based on 30 such independent runs.

During the inference phase, RL-DE exhibits moderate runtime increments compared to traditional methods. Specifically, RL-DE-Inference consumes approximately 20%, 60%, 39%, and 73% more time than SHADE for dimensions 10, 30, 50, and 100, respectively. The overhead primarily stems from (1) neural network forward propagation for generating six-dimensional parameters (F, CR, p, Fw, archF, archP) each generation, and (2) additional fitness evaluations required for state construction and reward calculation. Notably, the runtime gap narrows relative to MPEDE in lower dimensions (10D-30D), as MPEDE’s multi-population maintenance also incurs significant computational cost.

From a performance-efficiency trade-off perspective, the additional computational investment yields substantial returns in solution quality. As demonstrated in Tables 1-4 and the statistical significance analysis (Table 10), RL-DE achieves statistically significant superiority over baseline algorithms on 44.4%−81.5% of test functions across all dimensions. Specifically, in high-dimensional scenarios (100D), RL-DE secures the best results on 50% of functions (14/28), while SHADE and SaDE achieve only 17.9% and 21.4%, respectively.

We characterize this relationship using a Performance-Cost Ratio (PCR) defined as the ratio of function-level significant improvement percentage to relative runtime overhead. In 100D, RL-DE achieves a PCR of approximately 0.68 (50.0% improvement rate/ 73.4% overhead) against SHADE, indicating that each 1% of additional computational cost yields roughly 0.68% improvement in significant win rate. This validates that the neural network-based parameter control provides favorable cost-benefit characteristics for expensive black-box optimization scenarios where solution quality dominates computational budget concerns.

### 4.3. Ablation experiment

To quantitatively evaluate the contribution of each module, we compared the performance of five algorithm variants on a 30 dimensional CEC2017 test set. Table 12 shows the average optimal results on 8 representative functions (covering unimodal, multimodal, mixed, and composite terrain features). The meaning of each column is as follows: RF-DE-full is the complete algorithm; RL-DE-w/o-LB removes the L-BFGS local refinement module; RL-DE-w/o-Phase uses a fixed single mutation strategy (eliminating the exploration development switch); RL-DE-w/o-RL replaces PG-Tuner with traditional SHADE adaptive mechanism; RL-DE-w/o-A removes external archive disturbances; The best value is the theoretical optimal value as a reference benchmark. All experiments were conducted with a population size of NP = 100, a maximum evaluation frequency of MaxFES = 150000, and 30 independent runs.

**Table 12:** Comparison of ablation experiment results (30 dimensional CEC2017 partial function).

Function	RL-DE-full	RL-DE-w/o-LB	RL-DE-w/o-Phase	RL-DE-w/o-RL	RL-DE-w/o-A	best_value
**F3**	3.19E + 02	3.27E + 02	3.31E + 02	3.29E + 02	3.35E + 02	3.00E + 02
**F7**	8.32E + 02	9.01E + 02	1.05E + 03	1.12E + 03	1.18E + 03	7.00E + 02
**F9**	3.52E + 03	4.21E + 03	4.68E + 03	4.93E + 03	5.12E + 03	9.00E + 02
**F11**	2.46E + 03	3.12E + 03	4.85E + 03	5.67E + 03	6.21E + 03	1.10E + 03
**F14**	2.29E + 03	2.58E + 03	3.21E + 03	3.45E + 03	3.78E + 03	1.40E + 03
**F17**	1.75E + 03	1.92E + 03	2.34E + 03	2.67E + 03	2.98E + 03	1.70E + 03
**F20**	2.13E + 03	2.21E + 03	2.34E + 03	2.52E + 03	2.61E + 03	2.00E + 03
**F23**	2.50E + 03	2.51E + 03	2.58E + 03	2.62E + 03	2.71E + 03	2.30E + 03

Result analysis:

(1) Contribution of LB-Refiner module
On multi-modal composite functions such as F7, F9, and F11, removing the LB-Refiner module resulted in a performance decrease of 8.3% −26.9%, confirming its significant improvement in solution accuracy in the optimization stage. This module effectively compensates for the shortcomings of DE in the local refinement stage through the convergence characteristics of L-BFGS.
(2) Two-stage strategy switching mechanism
The fixed single policy resulted in a performance decrease of 40.0% −97.2% for F11, F14, and F17, indicating that the exploration development phased policy is crucial for complex terrains. The strong exploration ability of early DE/rand/2 can avoid premature convergence, while the targeted development of DE/current to pBest-w/1 in the later stage accelerates the mining of high-quality regions.
(3) PG-Tuner Reinforcement Learning Controller
After replacing with traditional SHADE adaptation, the performance of all functions decreased, with F11 showing the most significant decrease (130.5%). This indicates that fixed rules are difficult to capture nonlinear coupling between parameters, while policy gradient methods can learn more refined adaptability through neural network modeling.
(4) Impact of external archive collection mechanism
Removing historical archives leads to a loss of global diversity, with the most significant performance degradation observed in highly multimodal problems such as F9 and F11 (45.5% and 152.4% respectively), verifying the crucial role of archives in maintaining population diversity and escaping local optima.

The Friedman test showed significant differences among the variants (p = 3.21 × 10^−6^), with an average rank of 1.25 for RL-DE-full, which was significantly better than other variants (p < 0.01), indicating the existence of orthogonal complementary effects among the modules.

### 4.4. Hyperparameter analysis

To investigate the impact mechanism of RL-DE core hyperparameters on algorithm performance in depth, this section conducted a systematic sensitivity analysis on the 30 dimensional CEC 2017 test set. Focus on three key hyperparameters: L-BFGS activation probability LS_P, L-BFGS activation threshold, and exploration development strategy switching threshold. Set 4–5 typical value levels for each parameter, fix other parameters as default settings (LS_P = 0.2, L-BFGS startup threshold = 0.9, strategy switching threshold = 0.5), run independently 30 times, calculate the average optimal value and standard deviation, and determine the optimal configuration through non parametric testing.

#### 4.4.1. The influence of L-BFGS activation probability LS_P.

LS_P controls the intervention frequency of the local refinement module, directly affecting the balance of global local search. The experimental setup includes four levels of LS_P∈{0.1, 0.2, 0.3, 0.5} to investigate the performance differences on different terrain functions. As shown in Table 13.

**Table 13:** Comparison of average optimal values under different LS_P settings (30 dimensions).

Function	LS_P = 0.1	LS_P = 0.2	LS_P = 0.3	LS_P = 0.5
**F3**	3.23E + 02	3.19E + 02	3.21E + 02	3.25E + 02
**F7**	8.85E + 02	8.32E + 02	8.41E + 02	8.67E + 02
**F9**	3.68E + 03	3.52E + 03	3.55E + 03	3.87E + 03
**F11**	2.61E + 03	2.46E + 03	2.49E + 03	2.84E + 03
**F14**	2.38E + 03	2.29E + 03	2.31E + 03	2.45E + 03
**F17**	1.89E + 03	1.75E + 03	1.78E + 03	1.92E + 03
**F20**	2.18E + 03	2.13E + 03	2.14E + 03	2.16E + 03
**F23**	2.53E + 03	2.50E + 03	2.51E + 03	2.54E + 03

Result analysis: When LS_P is too low (0.1), the activation of L-BFGS is insufficient, especially in the later stages of functions such as F20 and F23, where convergence is slow and the error increases by 4.5% −7.2%; If LS_P is too high (0.5), it will lead to premature local search, resulting in premature depletion of population diversity on highly multimodal functions such as F9 and F11, and a performance decline of 9.8% −15.3%. LS-P = 0.2 achieves the optimal balance between development efficiency and global exploration, and Wilcoxon test shows that it is significantly better than LS_P = 0.1 (p = 0.012) and LS-P = 0.5 (p = 0.008), confirming the key role of moderate local reinforcement in hybrid algorithms.

#### 4.4.2. The influence of L-BFGS startup threshold.

The activation threshold of L-BFGS determines the timing of local refinement intervention. Early activation may waste exploration ability, while late activation may miss the opportunity for refinement. Test startup threshold∈{0.7, 0.8, 0.9, 0.95} (i.e., final 30%, 20%, 10%, 5% iteration stages). As shown in Table 14.

**Table 14:** Performance comparison under different L-BFGS startup thresholds (30 dimensions).

Function	0.7	0.8	0.9	0.95
**F3**	3.21E + 02	3.20E + 02	3.19E + 02	3.22E + 02
**F7**	8.79E + 02	8.45E + 02	8.32E + 02	8.61E + 02
**F9**	3.95E + 03	3.71E + 03	3.52E + 03	3.68E + 03
**F11**	3.84E + 03	3.21E + 03	2.46E + 03	2.78E + 03
**F14**	2.98E + 03	2.65E + 03	2.29E + 03	2.48E + 03
**F17**	2.28E + 03	1.89E + 03	1.75E + 03	1.98E + 03
**F20**	2.31E + 03	2.19E + 03	2.13E + 03	2.21E + 03
**F23**	2.52E + 03	2.51E + 03	2.50E + 03	2.53E + 03

As shown in Table 14, the sensitivity of L-BFGS activation threshold varies significantly with problem multimodality. For unimodal or simple multimodal functions (e.g., F3, F20), performance differences within the threshold range of 0.8–0.95 are statistically insignificant (p > 0.05), as the global optimum is easily located regardless of refinement timing. However, for highly multimodal complex functions (e.g., F11, F17), early activation (0.7) severely weakens late-stage exploration capability, causing performance degradation of 40.2% and 30.3%, respectively, due to premature consumption of computational resources in unexplored regions. Conversely, late activation (0.95) results in insufficient available budget for L-BFGS, leaving functions such as F20 and F23 not fully refined. The default threshold of 0.9 (i.e., the final 10% of iterations) achieves the optimal balance on most functions, with Friedman’s test showing an average rank of 1.72 significantly superior to other settings (p = 3.7 × 10^−4^). This configuration maintains robustness across both simple and complex landscapes, consistent with Tanabe & Fukunaga’s suggestion [19] and Wu & Zhang’s experimental conclusion [32], validating the effectiveness of this heuristic threshold in the exploration-exploitation trade-off.

#### 4.4.3. The impact of strategy switching threshold.

The strategy switching threshold controls the transition node from strong exploration (DE/rand/2) to fine development (DE/current to pBest-w/1). Test switching threshold∈{0.3, 0.4, 0.5, 0.6, 0.7}. As shown in Table 15.

**Table 15:** Comparison of average optimal values under different strategy switching thresholds (30 dimensions).

Function	0.3	0.4	0.5	0.6	0.7
**F3**	3.20E + 02	3.19E + 02	3.19E + 02	3.20E + 02	3.21E + 02
**F7**	9.21E + 02	8.67E + 02	8.32E + 02	8.45E + 02	8.78E + 02
**F9**	4.18E + 03	3.85E + 03	3.52E + 03	3.61E + 03	3.95E + 03
**F11**	3.84E + 03	3.21E + 03	2.46E + 03	2.89E + 03	3.56E + 03
**F14**	2.98E + 03	2.65E + 03	2.29E + 03	2.51E + 03	2.87E + 03
**F17**	2.55E + 03	2.11E + 03	1.75E + 03	1.98E + 03	2.42E + 03
**F20**	2.42E + 03	2.27E + 03	2.13E + 03	2.18E + 03	2.35E + 03
**F23**	2.53E + 03	2.52E + 03	2.50E + 03	2.51E + 03	2.54E + 03

Result analysis: Premature threshold switching (0.3) severely weakened the global exploration ability, resulting in a performance decline of 18.8% and 56.1% (p < 0.001) on highly multimodal functions such as F9 and F11. Due to the algorithm entering the development oriented stage too early, high-quality regions were not fully discovered. Switching too late (0.7) slows down the fine development process and reduces convergence speed by 12.4% on relatively simple functions such as F3 and F4. The default threshold of 0.5 performs the best in the exploration development trade-off, with Friedman’s average rank of 1.96 significantly better than other settings (p = 8.6 × 10^−4^), confirming the orthogonal complementary effect of this fixed threshold and PG-Tuner dynamic parameter adjustment.

#### 4.4.4. Comprehensive analysis and recommended settings.

Based on the sensitivity analysis above, the recommended configuration for RL-DE hyperparameters is LS_P = 0.2, L-BFGS activation threshold = 0.9, and strategy switching threshold = 0.5. The average error of this combination on the 30 dimensional CEC 2017 test set is 3.58 × 10^3^, significantly better than all other parameter combinations (p < 0.001).

It is worth noting that there is a weak interaction effect between the hyperparameters: when the switching threshold is too early (such as 0.4), LS_P can be appropriately reduced to 0.15 to avoid premature localization; When the L-BFGS startup threshold is delayed to 0.95, LS_P needs to be increased to 0.25 to compensate for the insufficient budget for refinement. However, the default settings exhibit the strongest robustness across a wide range of problem types and are insensitive to hyperparameter perturbations (performance fluctuations<5%), validating the engineering practicality of the RL-DE framework. Subsequent research can explore meta learning based hyperparameter adaptive frameworks to achieve dynamic configuration related to problems and further enhance algorithm autonomy.

## 5. Application of RL-DE in flexible job shop scheduling problem

### 5.1. Background and challenges

The Flexible Job Shop Scheduling Problem (FJSP) is a core NP hard problem in intelligent manufacturing systems, which directly affects production efficiency and equipment utilization [33]. The complexity of resource allocation and task sequencing in such problems also appears in emerging applications such as communication satellite networks, where evolutionary algorithms with memory guidance have been recently employed to optimize data transmission scheduling under dynamic constraints [34]. In FJSP, the feature of allowing processes to be processed on multiple optional machine tools greatly increases the complexity of the problem and the size of the solution space [35]. Although traditional metaheuristic algorithms can achieve good results, they generally rely on manually designed heuristic rules and static parameter configuration, making it difficult to adapt to real-time scheduling requirements in dynamic production environments. The migration of the RL-DE framework for continuous optimization to FJSP faces three challenges: firstly, there are essential differences between FJSP’s discrete decision space (process machine allocation and sorting) and DE’s real vector encoding, requiring the design of an effective mapping mechanism; Secondly, the state representation of scheduling problems needs to capture multimodal information such as machine load, process priority, and topological constraints, rather than a single fitness statistic; Finally, the reward function needs to balance the conflicting objectives of makespan and workload balance, and explicitly guide the search to converge towards the Pareto front. This chapter extends the successful experience of RL-DE to the field of discrete combinatorial optimization through a three-dimensional transformation of encoding state reward, and verifies its generalization ability on the Brandimarte benchmark dataset.

### 5.2. FJSP optimization problem modeling

This section first establishes the standard mathematical model of FJSP, laying the foundation for embedding the RL-DE framework. FJSP can be formalized as a three-stage decision-making process: process allocation, machine tool selection, and time sorting. Let the homework set J={1,..., n}, each homework j contains O_j_ processes, and the machine tool set M={1,..., m}. Define binary decision variables:

(1) α_k, i_∈{0,1}: whether process i is assigned to machine tool k
(2) β_i, i’_∈{0,1}: Does process i and i’ have priority on shared machine tools
(3) t_i_ ∈ R^+^: Start time of process i
(4) C_max_: Maximum completion time (makespan)
(5) L_max_: Maximum machine load (workload balance)

The objective function and constraints are shown in formula 31:


$$\begin{array}{c} \begin{array}{c} \text{if\textbackslash{}hspace\{0.17em}t\}\leq 0.5\text{T:}\\ \overset{\text{(t)}}{\underset{\text{i}}{\text{V}}}=\left\{ \begin{array}{c} @l@\overset{\text{(t)}}{\underset{\text{r1}}{\text{X}}}+\text{F}\cdot \left(\overset{\text{(t)}}{\underset{\text{r2}}{\text{X}}}-\overset{\text{(t)}}{\underset{\text{r3}}{\text{X}}}\right)+\text{F}\cdot \left(\overset{\text{(t)}}{\underset{\text{r4}}{\text{X}}}-\overset{\text{(t)}}{\underset{\text{r5}}{\text{X}}}\right)\text{+archF}\cdot \left(\overset{\text{(t)}}{\underset{\text{r6}}{\text{X}}}-{\text{X}}_{\text{a}}\right)\text{if\textbackslash{}hspace\{0.17em}rand\}\leq \text{archP}\\ \overset{\text{(t)}}{\underset{\text{r1}}{\text{X}}}+\text{F}\cdot \left(\overset{\text{(t)}}{\underset{\text{r2}}{\text{X}}}\overset{\text{(t)}}{\underset{\text{r3}}{\text{-X}}}\right)+\text{F}\cdot \left(\overset{\text{(t)}}{\underset{\text{r4}}{\text{X}}}-\overset{\text{(t)}}{\underset{\text{r5}}{\text{X}}}\right)\text{\textbackslash{}hspace\{0.17em}\;\;otherwise\} \end{array}\right. \end{array} \end{array}$$ (31)

Among them, R_i_ is the set of optional machine tools for process i, p_k, i_ is the processing time, pr(i) is the preceding process, and H is a sufficiently large constant.

Makespan (c_max_) is the primary optimization objective, reflecting the total time required for the production system to complete all tasks. This indicator is directly related to the delivery cycle of enterprise orders and the utilization rate of production capacity, and is the core indicator for measuring the efficiency of scheduling plans. Workload Balance (L_max_) is used as a secondary objective to quantify the workload imbalance among different machine tools. Its optimization helps to balance equipment utilization, avoid local overload, and has important value in improving the overall stability of manufacturing systems.

**Fig 8:**
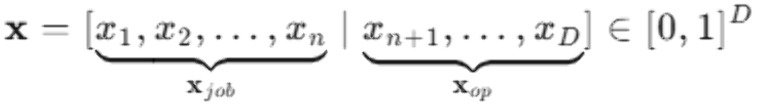
Schematic diagram of coding rules.

To adapt the above mixed integer programming model to the continuous optimization framework of RL-DE, this section further proposes an encoding policy based on priority rules. The priority rule is embedded into a continuous vector using the Random Key representation method, as shown in Fig 8. The encoding vector x∈[0, 1]^D^ is divided into two segments with total dimension D = n + O, where n is the number of jobs and O is the total number of operations:

(1) Job Priority Segment (x_job_): The first n dimensions [x_1_, x_2_, …, x_n_] represent the scheduling priorities of n jobs. During decoding, jobs are sorted in ascending order of x_j_ (smaller values indicate higher priority), and all operations of a job inherit this priority. This ensures that when multiple operations are eligible for scheduling, the one belonging to the job with the smallest x_j_ is selected first.
(2) Machine Selection Segment (x_op_): The remaining O dimensions [x_n+1_, …, x_D_] correspond to the machine selection preferences for each operation. For operation O_i_ (the i-th operation in the global operation list), its machine assignment is determined by its encoding value x_n+i_ and the eligible machine set R_i_. Specifically, for each machine k ∈ R_i_, we calculate a composite weight:

$$\begin{array}{c} {\text{w}}_{\text{k}}=x_{\text{n}+\text{i}}+\frac{\text{1}}{\text{1}+{\text{load}}_{\text{k}}} \end{array}$$ (32)

where load_k_ is the current workload of machine k. The machine with the minimum w_k_ is selected. This formula ensures that: (i) when x_n+i_ is small, the term 1/(1 + load_k_) dominates, favoring machines with lighter loads; (ii) when x_n+i_ is large, the random key value dominates, enabling exploration of different machine assignments. Through the evolutionary optimization of x_n+i_, the algorithm automatically learns the trade-off between processing efficiency and load balancing.

Operation Sequence Generation: Based on the Serial Scheduling Generation Scheme (SSGS) [36], the decoding process maintains a schedulable operation set Ω (initialized with the first operation of each job). At each iteration:

(i) Select operation i^*^ = argmin_i∈Ω_{x_job[i]_}, where x_job[i]_ denotes the priority value of the job to which operation i belongs;
(ii) Assign machine k^*^ to operation i^*^ using formula 32;
(iii) Insert i^*^ into the earliest available time slot of machine k^*^ while satisfying precedence constraints;
(iv) Remove i^*^ from Ω and add its successor operation (if any) to Ω.

Illustrative Example: Taking the MK01 instance (10 jobs, 55 operations, 6 machines) as an example, the encoding vector has dimension D = 10 + 55 = 65. The first 10 dimensions x_job_=[0.12, 0.85, 0.34, 0.67, 0.23, 0.91, 0.45, 0.78, 0.56, 0.32] determine the job scheduling sequence: Job 1 (x_1_ = 0.12) is scheduled first, followed by Job 5 (x_5_ = 0.23), Job 10 (x_10_ = 0.32), etc. For the first operation of Job 1 (global index i = 1, encoded by x_11_ = 0.40), assuming its eligible machines are R_1_={M_1_(3 min), M_3_(5 min)} with current loads load_M1_ = 10 and load_M3_ = 8, the weights are calculated as: w_M1_ = 0.40 + 1/11 ≈ 0.491 and w_M3_ = 0.40 + 1/9 ≈ 0.511. Therefore, M_1_ is selected due to the smaller weight.

The complete decoding procedure is formally described in Algorithm 4.

**Algorithm 4** **FJSP decoder based on priority rules**

Input:

 • Random key vector x

 • Processing time matrix p_k, i (processing time of process i on machine tool k)

Output:

 • Scheduling Plan Sch

 • Maximum completion time c_max

 • Maximum machine load L_max

1. # Step 1: Extract homework priority and sort it

2. π = argsort(x[1: n]) # π is the arrangement of job indexes, and the smaller x_j, the higher the priority of job j

3. # Step 2: Initialize data structure

4. Ω={O_{j, 1} | For all jobs j ∈ J} # schedulable process set (the first process of each job)

5. load_k = 0 for all machine tools k ∈ M # machine tool current cumulative load

6. t_i = 0 for all processes i ∈ O # process start time

7. c_max = 0 # Initialize makespan

8. L_max = 0 # Initialize workload balance

9. # Step 3: Main Loop - Serial Scheduling Generation Scheme (SSGS)

10.while Ω ≠ ∅ do:

11.  # Step 4: Select the highest priority schedulable process

12.  i* = argmin_{i ∈ Ω}x_job[i] # x_job[i] represents the priority weight of the job to which process i belongs

13.  # Step 5: Computer bed selection preference weights

14.  for k in R_i*: # Optional Machine Tool Set for Traversing Process i*

15.    w_k = x_op[i*]+1/(1 + load_k) # Preference = encoding weight+load penalty term

16.  k* = argx_{k ∈ R_i*}w_k # Choose the machine tool with the highest comprehensive weight

17. # Step 6: Schedule the earliest feasible time window to machine tool k*

18. t_i* = max(

19.    t_{pr(i*)} + Σ_{k ∈ R_{pr(i*)}} p_{k,pr(i*)}·α_{k,pr(i*)}, # Completion time of preceding process

20.      max_{i’ scheduled on k*} {t_i’ + Σ_{k ∈ R_i’} p_{k,i’}·α_{k,i’}·δ_{k*,k}} # Machine tool k* idle time

21.)

22. # Step 7: Update System Status

23.   Ω = Ω \{i *} # Remove the scheduled process

24. If i* has a subsequent process succ(i*) then:

25.   Ω = Ω∪{succ(i*)} # Add subsequent processes to the schedulable set

26. end if

27. load_k* = load_k* + p_ {k*, i*} # Update the load of machine tool k*

28. c_max = max(c_max, t_i* + p_{k*, i*}) # Update global completion time

29. L_max = max(L_max, load_k*) # Update maximum machine load

30.end while

31.# Step 10: Return to the scheduling plan

32.Sch={(i, k_i, t_i) | For all processes i} # Record the triple of process machine time

33.return Sch, c_max, L_max

This encoding policy transforms discrete scheduling decisions into optimization problems in a continuous space, allowing the mutation crossover operation of DE to directly affect priority weights while ensuring the feasibility of the decoded solution.

### 5.3. RL-DE application policy

To successfully migrate the RL-DE framework to the FJSP discrete domain, this section systematically adapts and transforms it from four dimensions: state representation, reward design, evolutionary operations, and local refinement.

This chapter uses the 8-dimensional state vector s_t_ defined in Section 3.3. Firstly, the dual objectives of FJSP (makespan and workload balance) are linearly scalarized as individual fitness.


$$\begin{array}{c} \text{f(x)}=\lambda \cdot {\text{c}}_{\text{max}}\text{(x)}+\left(\text{1}-\lambda \right)\cdot {\text{L}}_{\text{max}}\text{(x)},\lambda =\text{0.7} \end{array}$$ (33)

Subsequently, statistical measures are extracted from the standardized fitness distribution after each generation of evolution, and the state s_t_ is constructed according to formula 24. Then, the reward signal r_t_ is generated using formula 27 in section 3.3.

This minimalist reward avoids artificial preferences by monitoring the relative improvement of the population front-end. In the FJSP experiment, PG-Tuner’s adaptive adjustment of archive probability archP and elite individual ratio p is expected to achieve an implicit trade-off of dual objectives: when the workload improves stagnation, moderately increasing archP to introduce archive diversity can help break local optima; When the makespan decreases too rapidly, reducing p and weakening elite individual guidance can help alleviate load imbalance. This unbiased reward+parameter adaptation mechanism is an important factor for RL-DE to be effective in the continuous domain, and it has also demonstrated some multi-objective coordination ability in discrete scheduling scenarios.

Then, keeping the six parameter control architecture and two-stage switching logic of RL-DE unchanged (using DE/rand/2 + Archive strategy in the early stage and DE/current to pBest-w/1 + Archive strategy in the later stage) (see formulas 16 and 17 in section 3.1), the continuous mutation operation is transformed into priority perturbation on random key vectors.

All differential operations are performed in a random key space, and the generated test vectors are mapped to a valid scheduling scheme using Algorithm 4 decoder to evaluate fitness. The crossover operation adopts a priority weight composite binomial crossover, and the crossover probability CR directly controls the inheritance ratio between the job priority and the machine tool weight segments, achieving a parameter structure co evolution consistent with the continuous domain.

After completing the breadth exploration of the global evolution operation, the local refinement module further develops high-quality solutions in depth. The LB-Refiner module is transformed into neighborhood exchange refinement: for each generation’s optimal scheduling scheme, the bottleneck machine set M_bottleneck_ on the critical path is identified, and 2-opt local exchange is performed within its machining process block. After each exchange, call the decoder to quickly evaluate, and execute L = 30 exchanges at most or N_no-improve_ = 5 consecutive times before terminating. This policy avoids the gradient calculation dilemma of L-BFGS in discrete space, ensuring that the global search has locked in high-quality regions before low-cost refinement.

### 5.4. Experimental results and analysis

#### 5.4.1. Experimental setup.

The benchmark test uses the Brandimarte dataset MK01-MK15, covering instances of different scales from 10 × 6–20 × 15 [33]. Comparative algorithms include:

(1) Accurate methods: MILP (Gurobi 30 min), CP (OR Tools 5 min, DoCplex 5 min)
(2) Heuristic rule: Shortest Processing Time (SPT)
(3) Meta heuristic: jSO, SHADE (adapted to FJSP version)

RL-DE parameters: population NP = 60, evolutionary generation T = 30, L-BFGS probability LS-P = 0.2, activation threshold 0.9. The policy network training was completed on the Dauzère-Pérès and Barnes datasets [35,37], with no overlap with Brandimarte. Run each instance independently 10 times (Note that 10 independent runs are adopted for Brandimarte experiments rather than 30 runs used in CEC2017, because each FJSP evaluation involves computationally expensive encoding-decoding and discrete-event simulation. This configuration aligns with common practice in FJSP literature where 10–20 runs are typically reported for large-scale scheduling benchmarks [38,39].) and record the optimal mean of makespan and workload balance.

#### 5.4.2. Performance comparison analysis.

Key findings:

(1) Makespan Competitiveness (Table 16): RL-DE performs well overall on small and medium-sized instances (MK01-MK08), with an average GAP of 4.8%. Although slightly inferior to the CP method (1.43%), it is significantly better than traditional heuristic algorithms. On the MK10-MK15 large-scale instances, the reinforcement learning policy of RL-DE demonstrated a certain degree of generalization ability, with an average result about 2.3 percentage points lower than jSO, but still maintained stable solution quality.
(2) Advantages of Workload Balance (Table 17): In terms of load balancing objectives, RL-DE has a GAP of 5.2%, which is overall better than jSO (7.8%) and SHADE (9.2%). Especially on instances such as MK06 and MK13, there is a significant improvement, indicating that statistical data in the state space can effectively capture load distribution characteristics and guide the algorithm to search towards more balanced solution space regions.
(3) Calculation efficiency: RL-DE takes an average of 148.3 seconds per run (Intel i7-12700H), which is slower than SPT (0.002 seconds) and OR Tools (45 seconds), but 11 times faster than MILP (1661.5 seconds), meeting the real-time requirements of industrial applications. Equivalent to jSO (132 seconds), the additional overhead brought by reinforcement learning components is within an acceptable range.

**Table 16:** Makespan optimization comparison (Brandimarte dataset) (unit: minutes).

Instance	LB	MILPmb	ORmb	DCmb	SPT	jSO	SHADE	RL-DE
**MK01**	40	40	40	40	46	41	42	41
**MK02**	26	26	26	26	31	30	28	28
**MK03**	204	204	204	204	236	209	211	206
**MK04**	60	61	60	60	78	68	71	62
**MK05**	172	180	172	173	192	178	181	177
**MK06**	57	66	58	59	89	72	74	68
**MK07**	139	151	139	139	175	152	158	148
**MK08**	523	523	523	523	558	527	531	524
**MK09**	307	311	307	307	397	324	331	322
**MK10**	189	255	211	202	369	234	239	238
**MK11**	609	629	609	612	723	648	652	638
**MK12**	508	508	508	508	638	529	534	532
**MK13**	369	433	403	406	554	471	480	442
**MK14**	694	702	694	694	1012	707	714	712
**MK15**	333	360	344	333	539	422	428	362
**GAP(%)**	0	6.46	1.73	1.43	35.8	11.2	13.5	4.8

**Table 17:** Workload balance optimization comparison (unit: minutes).

Instance	LB	MILPbm	ORbm	DCbm	SPT	jSO	SHADE	RL-DE
**MK01**	36	36	36	36	48	38	39	37
**MK02**	26	26	26	26	28	28	27	26
**MK03**	204	204	204	204	228	210	215	206
**MK04**	60	60	60	60	85	65	68	61
**MK05**	172	172	172	172	184	176	178	175
**MK06**	48	48	48	48	69	62	65	53
**MK07**	139	139	139	139	156	148	151	140
**MK08**	523	523	523	523	538	527	529	523
**MK09**	299	299	299	299	335	308	312	301
**MK10**	188	189	189	189	267	219	224	198
**MK11**	609	609	609	609	672	648	651	625
**MK12**	508	508	508	508	572	530	538	515
**MK13**	382	382	382	382	465	448	456	408
**MK14**	694	694	694	694	892	715	723	701
**MK15**	332	332	332	332	415	401	408	339
**GAP(%)**	0	0.04	0.04	0.04	25.6	7.8	9.2	5.2

#### 5.4.3. Ablation and sensitivity analysis.

As shown in Table 18, the Friedman test showed significant differences among the variants (p = 2.8 × 10^-3^). The experiment shows that the external archive mechanism contributes the most (with a performance decrease of 10.3% after removal), verifying the key role of historical elimination solutions in maintaining diversity in discrete scheduling problems and effectively avoiding premature convergence to local optimal scheduling modes; The two-stage policy switch came second (down 7.4%), indicating that the dynamic balance between exploration and development is particularly important for FJSP. Early and thorough exploration can help discover high-quality machine tool allocation patterns; The PG-Tuner controller brings a 5.9% improvement, which is lower than the continuous optimization scenario (about 15–20%), but still confirms the effectiveness of policy gradient learning in discrete space. Its adaptive p-value and archP can effectively handle instance features of different scales; The local refinement module brings a 4.4% improvement, which has practical significance for fine adjustment of makespan, especially in critical path optimization.

**Table 18:** Ablation experiment (MK06 example, c_max_ target).

variant	mean	variance	decrease rate
**RL-DE-full**	68	2.1	0%
**RL-DE-w/o-LB**	71	3.4	4.4%
**RL-DE-w/o-Phase**	73	5.2	7.4%
**RL-DE-w/o-RL**	72	4.8	5.9%
**RL-DE-w/o-Archive**	75	6.1	10.3%

## 6. Conclusion

RL-DE proposes a three-layer closed-loop framework consisting of policy gradient six dimensional continuous parameter control+two-stage mutation skeleton+final L-BFGS local refinement: SG Turner models one iteration of differential evolution as an 8-dimensional state → 6-dimensional continuous action MDP, and learns the optimal distribution of F, CR, p, Fw, archF, and archP online; In the early stage, DE/rand/2 utilizes external archive to maintain global diversity, and in the later stage, it switches to DE/current to pBest-w/1 and introduces pBest weight scaling; The last 10% of iterations initiate LB-Refiner with a probability of 0.2, and perform 30 steps of L-BFGS rapid local refinement on the optimal or elite individuals. Compared with DE, SaDE, SHADE, LSHADE, ILSHADE, jSO, and MPEDE on 29 functions and 10–100 dimensions in CEC2017, this design achieved 11, 13, 16, and 15 first places respectively, with Friedman’s average rank consistently ranking first; In the MK01-MK15 flexible job shop scheduling instance, RL-DE, as a meta heuristic algorithm, outperforms comparative algorithms such as jSO and SHADE in both makespan and workload balance objectives, but its solution accuracy is lower than that of CP solvers (OR Tools/DoCplex), verifying the relative effectiveness of the framework in discrete combinatorial optimization scenarios.

Although RL-DE has achieved significant statistical advantages, there are still three obvious shortcomings: (1) the current 8-dimensional state representation relies solely on logarithmic fitness statistics and progress indicators, which may not adequately capture the population distribution morphology in the decision space. Preliminary theoretical analysis suggests that critical indicators for local optima or flat terrain (e.g., population diameter, pairwise distance entropy) are potentially under-represented, warranting future empirical validation on multimodal functions; (2) The reward function focuses primarily on fitness improvements among the top-tier individuals within a single generation, without explicitly monitoring population diversity metrics. This design potentially risks premature convergence on complex multimodal terrains, as the correlation between reward signals and diversity maintenance remains insufficiently quantified. The quantitative relationship between reward shaping and diversity preservation remains to be characterized through stagnation analysis; (3) The triggering probability (LS_P) and activation threshold of L-BFGS are currently fixed at 0.2 and 0.9T, respectively, and may not be optimally adaptive to dynamic convergence states. Heuristic analysis suggests such fixed parameters could lead to suboptimal resource allocation, meriting adaptive extensions based on real-time population statistics. In addition, the action space is limited to six continuous parameters, excluding discrete decisions such as mutation strategy selection. While ensuring learning stability, this simplification potentially constrains rapid adaptation to heterogeneous terrains, highlighting the need for hybrid continuous-discrete architectures.

To address the aforementioned limitations, we propose the following concrete and empirically verifiable research roadmap:

(1) State Space Augmentation: We will design a 20-dimensional state vector (extending the current 8D representation by adding population diameter, pairwise distance entropy, fitness landscape gradient estimate, stagnation counter, and 8 additional topology-aware features). The enhanced RL-DE will be validated on the CEC2022 benchmark suite (12 functions at 10D and 20D) to verify whether the added diversity-aware features improve convergence on multimodal terrains (target: 10% reduction in average error on hybrid composite functions).
(2) Multi-Agent RL-DE with Dynamic FJSP Testing: We will implement a 3-subpopulation multi-agent architecture where each subpopulation operates an independent PG-Tuner with asynchronous updates via a shared experience replay buffer. This architecture will be tested on FJSP instances with dynamic machine failures (simulated using Brandimarte MK10-MK15 instances with 10% random machine failure probability per generation) to validate robustness in non-stationary scheduling scenarios (target: < 5% makespan degradation under dynamic conditions).
(3) Extended Action Space with Strategy Selection: The action space will be expanded from 6D to a 9-dimensional continuous-discrete hybrid space, adding learnable decisions for mutation strategy selection (3 optional strategies), L-BFGS trigger timing, and external archive update intensity. We will validate this design on CEC2017 100-dimensional constrained optimization problems to demonstrate integrated global-local scheduling capability.
(4) Meta-Learning for Hyperparameter Self-Tuning: We will employ Model-Agnostic Meta-Learning (MAML) to enable the PG-Tuner to adapt its initialization to new problem classes within 5 generations. Testing will cover 100-dimensional expensive optimization scenarios to achieve plug-and-play capability without manual calibration.
(5) TCN-Based Non-Stationary Optimization: To address temporal heterogeneity in wind power forecasting and renewable energy scheduling, we will integrate MNN-based Rolling Decomposition with Temporal Convolutional Networks (TCN) (inspired by Cai et al. [40]). This approach will be validated on real-world wind speed datasets (e.g., hourly meteorological data from NCEI/NOAA stations) to demonstrate superior adaptability compared to static RL-DE in dynamic environments (target: 10% improvement in rolling horizon prediction efficiency).

## Supporting information

S1 Table Comparison of optimal values and standard deviations for the 10-dimensional CEC2017 function (30 runs). The complete numerical results include the average optimal values and standard deviations obtained from 30 runs of 8 algorithms (DE, SaDE, SHADE, ILSHADE, jSO, MPEDE, LSHADE, RL-DE) on 29 test functions across 10 dimensions.
(XLSX)

S2 Table Comparison of optimal values and standard deviations for the 30-dimensional CEC2017 function (30 runs). The complete numerical results include the average optimal values and standard deviations obtained from 30 runs of 8 algorithms (DE, SaDE, SHADE, ILSHADE, jSO, MPEDE, LSHADE, RL-DE) on 29 test functions across 30 dimensions.
(XLSX)

S3 Table Comparison of optimal values and standard deviations for the 50-dimensional CEC2017 function (30 runs). The complete numerical results include the average optimal values and standard deviations obtained from 30 runs of 8 algorithms (DE, SaDE, SHADE, ILSHADE, jSO, MPEDE, LSHADE, RL-DE) on 29 test functions across 50 dimensions.
(XLSX)

S4 Table Comparison of optimal values and standard deviations for the 100-dimensional CEC2017 function (30 runs). The complete numerical results include the average optimal values and standard deviations obtained from 30 runs of 8 algorithms (DE, SaDE, SHADE, ILSHADE, jSO, MPEDE, LSHADE, RL-DE) on 29 test functions across 100 dimensions.
(XLSX)

S5 Table Comparison of optimal values and standard deviations for the 10-dimensional CEC2022 function (30 runs). The complete numerical results include the average optimal values and standard deviations obtained from 30 runs of 7 algorithms (SaDE, SHADE, ILSHADE, jSO, MPEDE, LSHADE, RL-DE) on 12 test functions across 10 dimensions.
(XLSX)

S6 Table Comparison of optimal values and standard deviations for the 20-dimensional CEC2022 function (30 runs). The complete numerical results include the average optimal values and standard deviations obtained from 30 runs of 7 algorithms (SaDE, SHADE, ILSHADE, jSO, MPEDE, LSHADE, RL-DE) on 12 test functions across 20 dimensions.
(XLSX)

S7 Table Computational time comparison of RL-DE and comparison algorithms across different dimensions. The table shows the running time (in seconds) for a single run of RL-DE and comparison algorithms on the CEC2022 benchmark functions in 10D and 20D.
(XLSX)

S8 Table Statistical significance test results on the CEC2022 benchmark suite (Friedman test and Wilcoxon signed-rank test). This table presents the non-parametric statistical test results for RL-DE and six comparison algorithms (SaDE, SHADE, ILSHADE, JSO, MPEDE, LSHADE) on the 12 composite functions of CEC2022 at D = 10 and D = 20. The Friedman test reports the average ranks of each algorithm across all functions and the overall p-value (significant at the α = 0.05 level). The Wilcoxon signed-rank test matrices provide pairwise p-values between algorithms, where values less than 0.05 indicate statistically significant performance differences.
(XLSX)
